# Orchestrated response from heterogenous fibroblast subsets contributes to repair from surgery-induced stress after airway reconstruction

**DOI:** 10.1172/jci.insight.186263

**Published:** 2025-01-21

**Authors:** Jazmin Calyeca, Zakarie Hussein, Zheng Hong Tan, Lumei Liu, Sayali Dharmadhikari, Kimberly M. Shontz, Tatyana A. Vetter, Christopher K. Breuer, Susan D. Reynolds, Tendy Chiang

**Affiliations:** 1 Department of Otolaryngology and; 2 Center for Regenerative Medicine, Abigail Wexner Research Institute at Nationwide Children’s Hospital, Columbus, Ohio, USA.; 3 The Ohio State University College of Medicine, Columbus, Ohio, USA.; 4 Center for Gene Therapy, Abigail Wexner Research Institute, and; 5 Center for Perinatal Research, Nationwide Children’s Hospital, Columbus, Ohio, USA.; 6 Department of Otolaryngology - Head and Neck Surgery, The Ohio State Wexner Medical Center, Columbus, Ohio, USA.

**Keywords:** Cell biology, Transplantation, Bioinformatics, Fibrosis, Surgery

## Abstract

Surgery of the tracheobronchial tree carries high morbidity, with over half of the complications occurring at the anastomosis. Although fibroblasts are crucial in airway wound healing, the underlying cellular and molecular mechanisms in airway reconstruction remain unknown. We hypothesized that airway reconstruction initiates a surgery-induced stress (SIS) response, altering fibroblast communication within airway tissues. Using single-cell RNA-Seq, we analyzed native and reconstructed airways and identified 5 fibroblast subpopulations, each with distinct spatial distributions across anastomotic, submucosal, perichondrial, and paratracheal areas. During homeostasis, adventitial and airway fibroblasts (Adventitial-Fb and Airway-Fb, respectively) maintained tissue structure and created cellular niches by regulating ECM turnover. Under SIS, perichondrial fibroblasts (PC-Fb) exhibited chondroprogenitor-like gene signatures, and immune-recruiting fibroblasts (IR-Fb) facilitated cell infiltration. *Cthrc1*-activated fibroblasts (*Cthrc1^+^* Fb), mainly derived from Adventitial-Fb, primarily contributed to fibrotic scar formation and collagen production, mediated by TGF-β. Furthermore, repeated SIS created an imbalance in fibroblast states favoring emergence of *CTHRC1^+^* Fb and leading to impaired fibroblasts–basal cell crosstalk. Collectively, these data identify PC, IR, and *Cthrc1*^+^ Fb as a signaling hub, with SIS emerging as a mechanism initiating airway remodeling after reconstruction that, if not controlled, may lead to complications such as stenosis or anastomotic breakdown.

## Abstract 2

This study identifies fibroblasts as a signaling hub in airway repair, uncovers their heterogeneity and distinct spatial distribution, and elucidates their specific roles in airway reconstruction.

## Graphical abstract

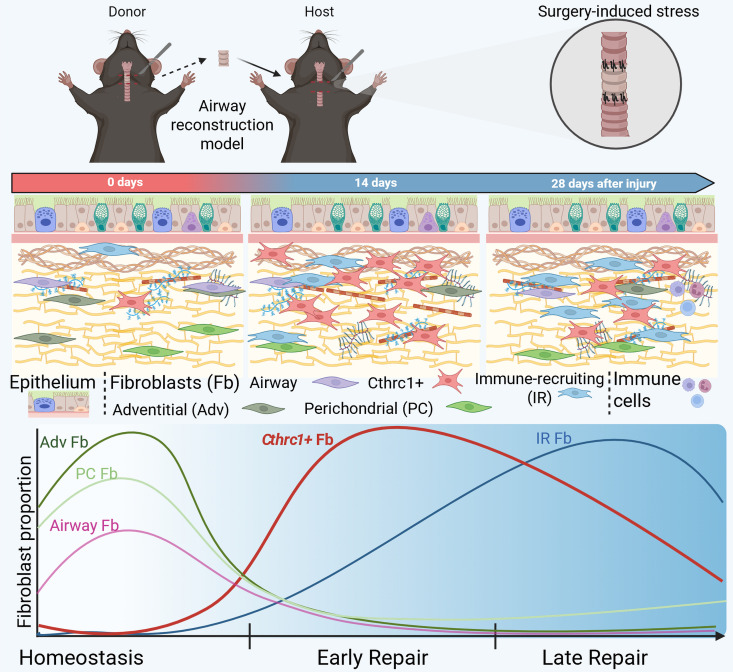

## Introduction

Tracheobronchial surgery encompasses a diverse set of interventions for the management of large airway conditions including airway stenosis, neoplasm, injury, and lung transplantation (1). A common indicator of positive outcomes after tracheobronchial surgery is the effective healing of an end-to-end anastomosis. However, airway anastomotic complications are common in large airway surgery, occurring in approximately 1.4%–33% of lung transplant cases, resulting in substantial morbidity and mortality (2, 3). Despite advancements in surgical technique and postoperative management, the optimal approach for managing these complications remains elusive.

During the healing process, early anastomotic complications, including dehiscence, are few (1%) but deadly (4–6); late anastomotic complications predominantly manifest as stenosis and occur in 1.6–32% of lung transplant cases (3, 7). Stenosis is characterized by impaired epithelial regeneration and progressive fibrosis (8), culminating in airway narrowing, impaired airflow, and diminished respiratory capacity (1). Although recent studies suggest that fibrosis and stenosis of the postsurgical airway is a consequence of aberrant wound healing, extracellular matrix (ECM) remodeling (9), and compromised immune responses (10), the precise orchestrating mechanisms remain unknown.

Accumulated evidence supports the pivotal role of fibroblasts (Fb) in airway repair, as well as their involvement in the pathogenesis and progression of fibrosis (11, 12). However, the majority of studies in anastomotic healing have centered on resident Fb as a unique population characterized by the expression of prespecified markers (13) or as a cell type that is primarily limited to the synthesis of ECM components (14, 15). The advent of single-cell RNA-Seq (scRNA-Seq) has altered this paradigm, revealing that Fb are a morphologically and functionally heterogeneous population. This diversity has emerged as a critical aspect of diseases characterized by aberrant wound healing processes in organs such as lung, heart, and skin (16–18), thus offering profound insights into the intricate interplay among Fb, epithelial, and immune cells and the notion that a better understanding of these interactions will guide the development of therapeutic strategies aimed at mitigating tissue fibrosis (19, 20).

Previously, we demonstrated that airway anastomosis encompasses changes in the immune milieu (21), activates airway basal stem cells, shifts the distribution of epithelial cell subtypes (22), and remodels the ECM (23). In the present study, our objective is to delineate cellular hubs and interactions involved in anastomotic repair through the integration of scRNA-Seq and multiplex immunofluorescence approaches. We used a long-term survival preclinical model of segmental airway reconstruction to determine how the cellular composition of the graft responds to surgical stress and characterized changes in their specific gene expression profiles in the absence of chronic airway disease. Our findings revealed the presence of distinct Fb subpopulations, creating the airway niche during repair. We found that the surgical trauma triggers a stress response (surgery-induced stress [SIS]) that acts on homeostatic Fb and influences them to differentiate through transitional states. Evidence of Fb contribution to airway repair following reconstruction involves differentiation to chondrocytes, orchestration of immune response, and regulation of collagen production/remodeling. Specifically, we identified the presence of a *Cthrc1^+^* Fb population as the major contributor to the remodeled ECM, particularly through changes in collagen composition. Mechanistically, we identified that SIS increased TGF-β receptor expression in *Cthrc1^+^* Fb, and inhibition of the TGF-β pathway abrogated Fb activation, CTHRC1 expression, and collagen synthesis in Airway-Fb. We conclude that a response to SIS involves a coordinated emergence of distinct Fb subsets participating in airway repair.

## Results

### SIS results in Fb accumulation in the reconstructed airway.

**Figure 1:**
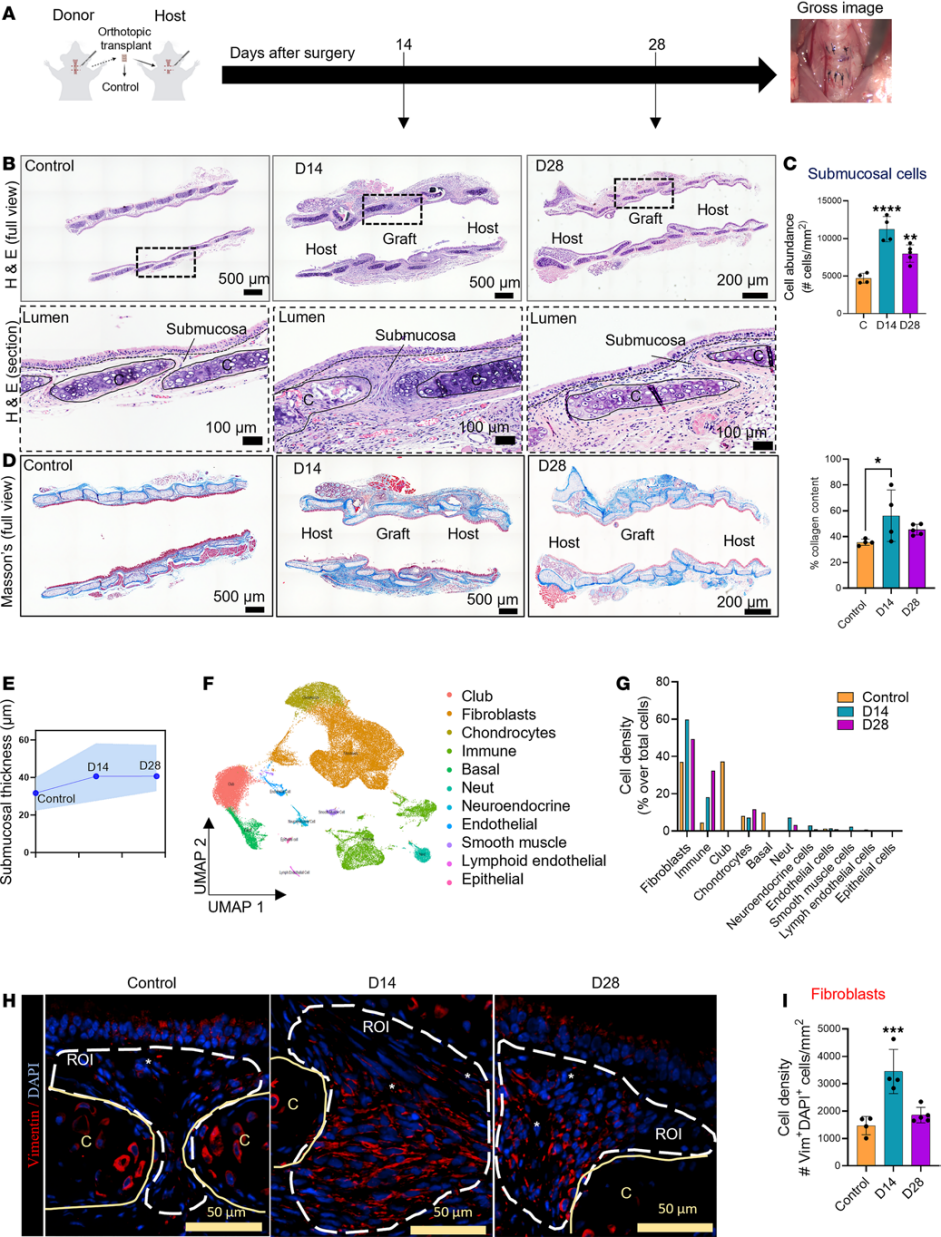
Surgery-induced stress results in fibroblast accumulation in the reconstructed airway. (**A**) Graphical representation of the microsurgical model of airway reconstruction with a corresponding gross image showing the site of surgery. (**B**) Representative full and section view of H&E images of homeostatic and reconstructed airways at D14–D28. (**C**) Quantification of the number of cells per mm^2^ square in the submucosa (Control, *n* = 4; D14, *n* = 4; D28, *n* = 5). (**D**) Masson’s trichrome staining shows the luminal surface, epithelium, submucosa area, and cartilage (Control, *n* = 4; D14, *n* = 4; D28, *n* = 5). (**E**) Quantification of submucosa thickness at different time points following reconstruction. Dots represent the mean, and colored area represents upper and lower limits (Control, *n* = 4; D14, *n* = 4; D28, *n* = 5). (**F**) UMAP and annotation of all scRNA-Seq cells from the normal airway, and D14 and D28 after surgery (*n* = 63,561 cells). (**G**) The proportion of cell lineages in the normal airway and D14 and D28 after reconstruction. (**H**) Representative immunofluorescence staining showing the presence of fibroblasts (Vimentin^+^, red). (**I**) Quantification of Fb per mm^2^ (Vimentin^+^, red) per condition (Control *n* = 4, D14 *n* = 4, D28 *n* = 5). For all staining, “C” indicates cartilage, and the dotted line indicates the epithelium. Data are shown as mean ± SD. Statistical analysis was performed using 1-way ANOVA with multiple-comparison test. *P < 0.05, **P < 0.01, ***P < 0.001, and ****P < 0.0001. Scale bars: 50 µm, 100 µm, 200 µm, and 500 µm as indicated.

In our previous work, we showed that ECM turnover and remodeling are affected after airway reconstruction, leading to alterations in the matrisome (23). While ECM remodeling is a normal aspect of wound healing, a dysregulated process might result in pathological scarring concomitant with fibrosis (24). To evaluate ECM remodeling in response to SIS, we developed a preclinical model of airway reconstruction in which a tracheal segment was orthotopically transplanted into a genetically identical host (25). We first investigated the degree to which surgery alters airway homeostasis by examining histological changes in the operative site at early (postoperative day 14 [D14]) and late (D28) time points (Figure 1A). In homeostasis (native trachea hereafter referred to as Control), the epithelial submucosa, the region between the tracheal cartilage and epithelium, is mostly comprised of an acellular matrix, blood vessels, and scarce Fb (Figure. 1B). After reconstruction, evidence of SIS included active remodeling (Figure 1B) and increased cellular infiltration (Figure 1C). The SIS response included increased signs of fibrosis, as evidenced by acute collagen deposition at D14 with spontaneous resolution of this change by D28 (Figure 1D). Changes in the submucosa were also reflected by an increase in submucosal thickness (Figure 1E).

To investigate the cellular aspects of SIS response, Control (native trachea) and SIS (reconstructed tracheas, D14 and D28) transcriptomes (*n* = 63,561 cells) from our recently published scRNA-Seq data set (22) were reanalyzed and allowed us to map the cells that were infiltrating the airway niche in response to surgery. Unsupervised computational analysis identified 20 discrete clusters corresponding to 11 cell types based on their distinct markers (Figure 1F and Supplemental Figure 1A; supplemental material available online with this article; https://doi.org/10.1172/jci.insight.186263DS1). As shown in Figure 1G, SIS altered the cellular composition within the airway niche, where in contrast to the native trachea, the most abundant cells found in postreconstructed airway were Fb. We performed immunostaining in homeostatic and reconstructed airways and confirmed a significant increase of Fb (Vimentin) within the submucosa in response to surgery (Figure 1, H and I). Together these results demonstrated that SIS altered the airway cellular niche, favoring Fb emergence and accumulation.

### Classification of Fb heterogeneity by scRNA-Seq reveals 5 subpopulations.

**Figure 2:**
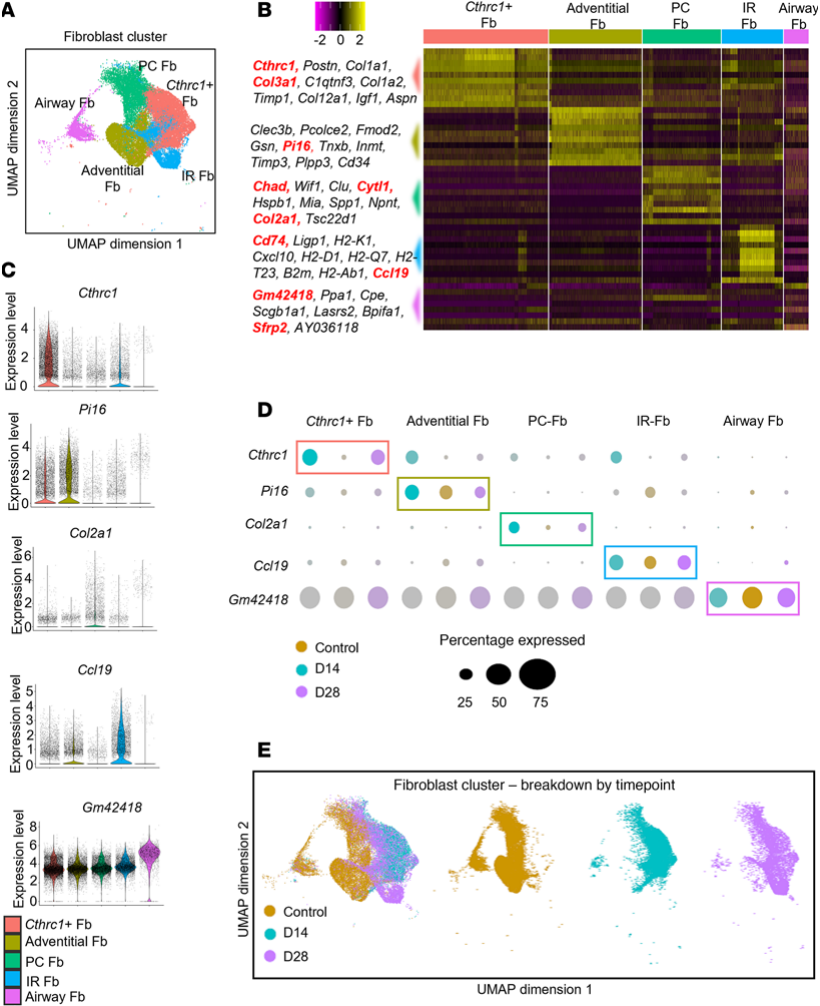
Airway fibroblast heterogeneity identified by scRNA-Seq. (**A**) UMAP and annotation displaying fibroblast subclustering in 5 subpopulations: Airway-Fb, Adventitial-Fb, PC-Fb, CP-Fb, and IR-Fb (*n* = 28,808 Fb). (**B**) Heatmap showing the 10 most differentially expressed genes of each fibroblast cluster, as provided by Seurat. Each column represents a single cell, and each row represents an individual gene. All marker genes per cluster are shown on the left. Yellow indicates maximum gene expression, and purple indicates no expression in scaled log normalized UMI counts. (**C**) Violin plots showing representative expressed genes among Fb subtypes. (**D**) Dot plot showing the expression of the top marker of each fibroblast subset among conditions. (**E**) Fb cells in UMAP are colored by their origin: normal airway and D14 and D28 after reconstruction.

Next, we evaluated whether these Fb constitute a uniform or a transcriptionally distinct cell population. We first identified all stromal cell populations (Supplemental Figure 2A) and removed cells such as smooth muscle cells (*Acta2*) and chondrocytes (*Col2a1*, *Sox9*). Using this strategy and Uniform Manifold Approximation and Projection (UMAP) visualization, we identified 5 discrete clusters within the Fb subset of native and reconstructed airways (*n* = 28,808 Fb) (Figure 2A and Supplemental Figure 2B). By cross-referencing differentially expressed genes in each cluster with previously published cell type–specific markers and manual annotation, these were named: *Cthrc1^+^* Fb (Cluster 1: *Cthrc1*, *Postn*, *Col1a1*, *Col3a1*, *C1qtnf3*), Adventitial-Fb (Cluster 2: *Clec3b*, *Pcolce2*, *Fmod2*, *Gsn*, *Pi16*), perichondral Fb (PC-Fb) (Cluster 3: *Chad*, *Wif1*, *Clu*, *Cytl1*, *Hspb1*), immune-recruiting Fb (IR-Fb) (Cluster 5: *Cd74*, *Ligp1*, *H2-K1*, *Cxcl10*, *H2-D1*), and Airway-Fb (Cluster 9: *Gm42418*, *Ppa1*, *Cpe*, *Scgb1a1*, *Lasrs2*) (Figure 2B). Each subpopulation of Fb had a characteristic gene expression profile (Figure 2, C and D, and Supplemental Figure 2C). Comparing the Fb cluster by time points revealed transient shifts in Fb gene signatures in response to SIS (Figure 2E). Together, these findings indicate that Airway-Fb constitute a heterogeneous population, with distinct transcriptomic signatures during homeostasis and repair.

### SIS selectively alters Fb proportions.

**Figure 3:**
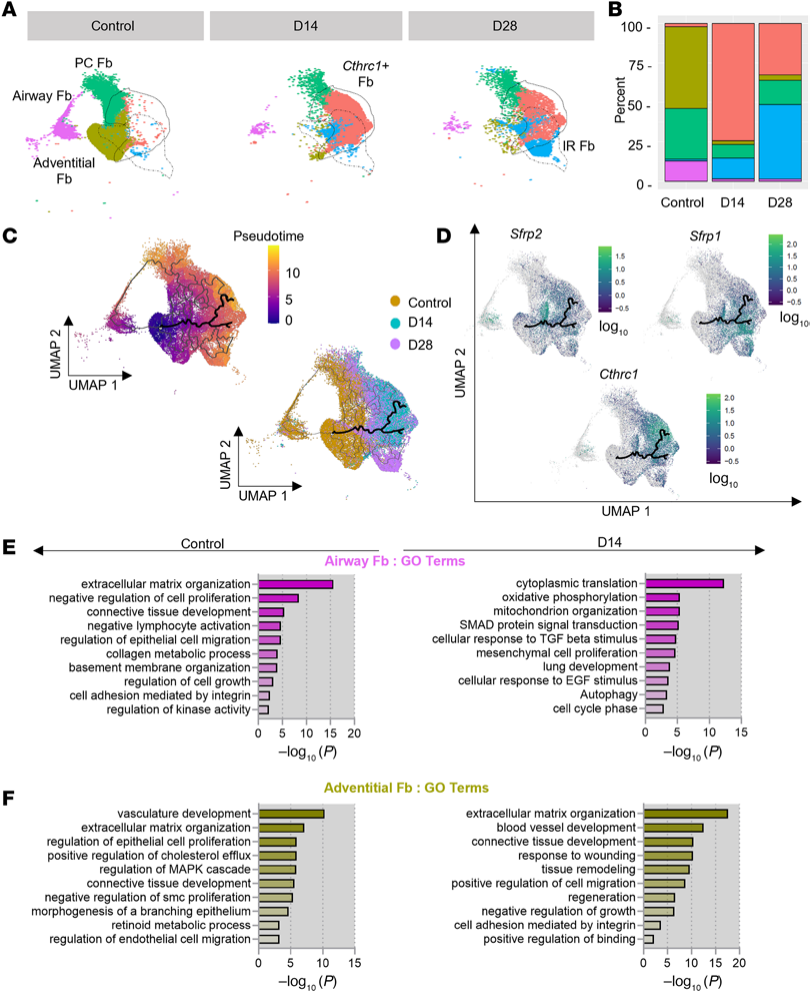
Surgery-induced stress selectively alters subtypes proportion of the fibroblast landscape in response to early and late repair events. (**A**) Fb clusters in UMAP are colored by their subtype from the normal airway and D14 and D28 after reconstruction. (**B**) The proportion of Fb subtypes in the normal airway and D14 and D28 after reconstruction. (**C**) Monocle analysis of Fb subsets trajectory as indicated by the black lines, with cells colored by pseudotime (upper panel) or their origin: normal, D14, and D28 (lower panel). (**D**) UMAP shows the expression levels of *Sfrtp2*, *Sfrtp1*, and *Cthrc1* in pseudotime. *Sfrp2* and *Sfrp1* were expressed more highly by cells clustered earlier in pseudotime, corresponding to Airway-Fb and Adventitial-Fb (left upper panel and right upper panel, respectively). *Cthrc1* was more highly expressed later in pseudotime, corresponding to CP-Fb identified in UMAP. (**E**) GO-enriched pathways corresponding to Airway-Fb during homeostasis and D14 after reconstruction (left panel and right panel, respectively). (**F**) GO-enriched pathways corresponding to Adventitial-Fb during homeostasis and D14 after reconstruction (left panel and right panel, respectively). FDR < 10%.

Since we identified 5 distinct Airway-Fb subpopulations, we then measured the effect of SIS on their abundance (Figure 3A). In homeostasis, we found that the most abundant Fb subtypes were adventitial (52%), followed by perichondrial (32%) and airway (13%) with immune-recruiting and *Cthrc1^+^* subtypes comprising scarce proportions (2%, 1% respectively). In contrast, during SIS both *Cthrc1^+^*-Fb (D14, 74%; D28, 33%) and IR-Fb (D14, 13%; D28, 47%) became the most abundant subpopulations with PC-Fb (D14, 9%; D28, 15%) Adventitial (D14, 2%; D28, 3%) and Airway (D14, 1%; D28, 1%) becoming the least abundant (Figure 3B).

Given the dramatic reduction in the Adventitial-Fb proportion, we used pseudotime analysis to assess the fate of this subpopulation during SIS. Adventitial-Fb have a potential capacity to differentiate toward a *Cthrc1^+^* phenotype in response to lung injury (26). Although in the lung *Cthrc1^+^* Fb emerge from alveolar Fb (27), in the airway, we found that Airway-Fb and Adventitial-Fb differentiated into a *Cthrc1^+^* cell state in response to SIS (Figure 3C). Overexpression of transitional state markers *Sfrp1* and *Sfrp2* demonstrated a high probability that these cell states are precursors of *Cthrc1^+^* activated Fb state (Figure 3D), which is consistent with previous studies (28, 29).

With the suspicion that Airway-Fb and Adventitial-Fb are transitioning to other Fb subtypes, specially to *Cthrc1^+^*, we further characterized differentiation reprogramming by SIS using gene ontology (GO) analysis. As expected for Fb during homeostasis, we found that ECM organization was one of the most enriched pathways (Figure 3, E and F) for both the airway and Adventitial-Fb and that they had similar expression of common transcription factor regulators *Cebpb*, *Pax6*, and *Sp1* (Supplemental Figure 3A). However, SIS rewired cellular processes such as oxidative phosphorylation and mitochondrion organization, cellular response to TGF-β stimulus, response to wounding, tissue remodeling, and regeneration to predominantly support differentiation (Figure 3, E and F). Collectively, these results suggest that SIS induces transcriptomic reprogramming of homeostatic Fb subpopulations into activated states.

### PC-Fb differentiate into chondrocytes after airway reconstruction.

**Figure 4:**
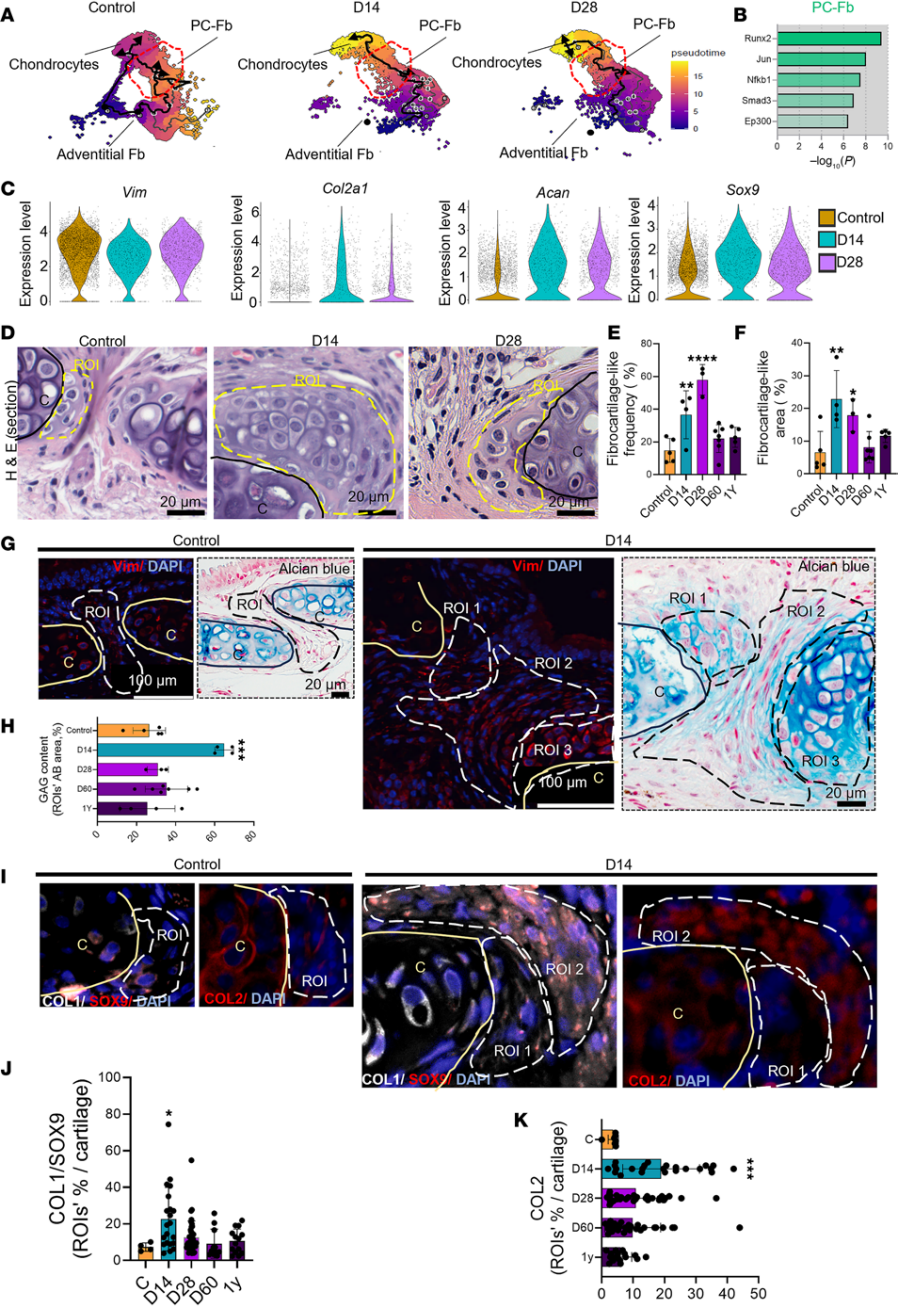
Perichondral fibroblasts modulate their identity to chondrocytes during repair. (**A**) Monocle analysis of Fb subsets trajectory as indicated by the black lines, with cells colored by pseudotime in normal airway (left panel), D14 (middle panel), and D28 (right panel). (**B**) Bar graph showing identified potential transcription factors in perichondral Fb at D14 compared with Control. (**C**) Violin plots showing representative expressed fibroblast or chondrocyte marker genes in the PC-Fb cluster during homeostasis and repair. (**D**) Representative H&E staining shows the fibrocartilage-like structure closed to the cartilage. (**E** and **F**) Quantification of fibrocartilage-like frequency (presence/cartilage per animal) (**E**) and area (**F**) (Control, *n* = 4; D14, *n* = 4; D28, *n* = 3; D60, *n* = 7; 1 year [Y]; *n* = 5). (**G**) Representative immunofluorescence and alcian blue staining from serial sections show ROI indicating Vimentin (red) and alcian blue^+^ cells between the cartilage and next to the cartilage. Region of interest (ROI) 1–3 reminiscent of PC-Fb at different stages toward the chondrocyte identity (left panels). (**H**) Quantification of fibrocartilage-like proteoglycan content in the normal airway and following reconstruction (Control, *n* = 4; D14, *n* = 4; D28, *n* = 3; D60, *n* = 7; 1Y, *n* = 5). (**I**–**K**) Representative images of immunofluorescence analysis of normal (right panels) and reconstructed airway sections (D14) (left panels) demonstrating the appearance of COL1/SOX9–double-positive cells (white/red) and COL2^+^ cells (red) and corresponding quantification of all conditions (Control, *n* = 4; D14, *n* = 4; D28, *n* = 3; D60, *n* = 7; 1Y, *n* = 5). For all staining, “C” indicates cartilage, and ROI dotted line indicates the fibrocartilage-like structure. Statistical analysis was performed using 1-way ANOVA with Dunnett’s multiple-comparison test. *P < 0.05, **P < 0.01, ***P < 0.001, and ****P < 0.0001. Scale bars: 20 µm and 100 µm as indicated.

Fb are a potential chondroprogenitor for repair of acutely damaged cartilage (30). In the trachea, previous evidence has identified PC-Fb as an intermediate state on the trajectory from Adventitial-Fb to chondrocyte (31). In our model of airway reconstruction, PC-Fb showed temporal changes to their proportion, starting with a reduction from homeostasis to D14 (32%–9%) with a modest increase to 15% by D28 (Figure 3, A and B). To further investigate the differentiation commitment in this subset, we conducted a trajectory analysis, and we found that during homeostasis, PC-Fb showed a similar probability of commitment to differentiate into chondrocytes or to undergo self-renewal (Figure 4A). However, after reconstruction, PC-Fb lost their self-renewal commitment and showed a higher probability of acquiring a chondrocyte identity (Figure 4A). This process was likely to be mediated by the chondrocyte maturation inducer (32) *Runx2* (Figure 4B). Violin plots of chondrocyte fate–associated genes confirmed relatively increased expression of *Col2a1*, *Acan*, *Sox9* (chondrogenic genes) and relative reduction of the Fb marker (*Vim*) by PC-Fb (Figure 4C) in response to SIS.

To further evaluate the implications of perichondrial Fb differentiation in vivo, we performed an exhaustive histologic evaluation of the reconstructed airway in a longitudinal study encompassing data from 1 year after surgery (Figure 4D and Supplemental Figure 4A). This analysis revealed the presence of fibrocartilage-like structures adjacent to the cartilage rings (Figure 4D), which appeared to be most prominent in size and frequency at D14 compared with D28. At D60, both frequency and size returned to normal and remained stable at 1 year (Figure 4, D–F, and Supplemental Figure 4A).

When we stained the fibrocartilage-like structures for glycosaminoglycans (GAG), a vital constituent of cartilage, and Fb markers (Vimentin), we found that these regions were in different states of differentiation, exclusively in groups following airway reconstruction. Particularly, we identified 3 different states; 1 composed of Fb-shaped cells and 2 other states composed of chondrocyte-shaped cells which differed based on ECM composition (Figure 4, G and H). We then quantified GAG content in the fibrocartilage-like structures. Mirroring the changes of size and frequency, fibrocartilage-like structures expressed more GAG at D14 compared with any other time point (Figure 4H and Supplemental Figure 4B). Pursuing the idea that fibrocartilage-like structures were still expressing both Fb and chondrocyte markers, we costained homeostatic and reconstructed airways for COL1 and SOX9, and we found that the double-positive area was significantly higher at D14 compared with other time points (Figure 4, I and J, and Supplemental Figure 4C). Similarly, COL2 area in fibrocartilage-like structures was highest at D14 (Figure 4, I and K, and Supplemental Figure 4D).

Taking into consideration that PC-Fb differentiation to chondrocytes occurs in response to cartilage damage (31), we subsequently evaluated the effect of SIS on cartilage/chondrocytes. μ-CT showed increased Hounsfield units of the airway cartilage at both postsurgical time points (D14 and D28) compared with homeostasis (Supplemental Figure 5, A and B), indicating increased cartilage calcification after reconstruction. At the transcriptomic level, evaluation of DEGs in the chondrocyte cluster revealed upregulated expression of hypertrophic remodeling (*Col11a1*, *Col3a1*, *Col1a1*, *Col9a1*) and stress response genes (*Hspb1*, *Hspa1a*, *Hspa1*, *Dnajb1*, *Cdkn1a*) (Supplemental Figure 5C) in response to SIS. At the functional level, GO analysis showed enrichment of pathways associated with cellular response to stress and cell cycle regulation (Supplemental Figure 5, C and D) controlled by the transcription factor *Cebpb* (Supplemental Figure 5E). Collectively, these findings indicate that perichondrial Fb form fibrocartilage-like structures, potentially differentiating into chondrocytes, most likely in response to mild cartilage damage.

### IR-Fb expansion coincides with immune cell recruitment and infiltration after reconstruction.

**Figure 5:**
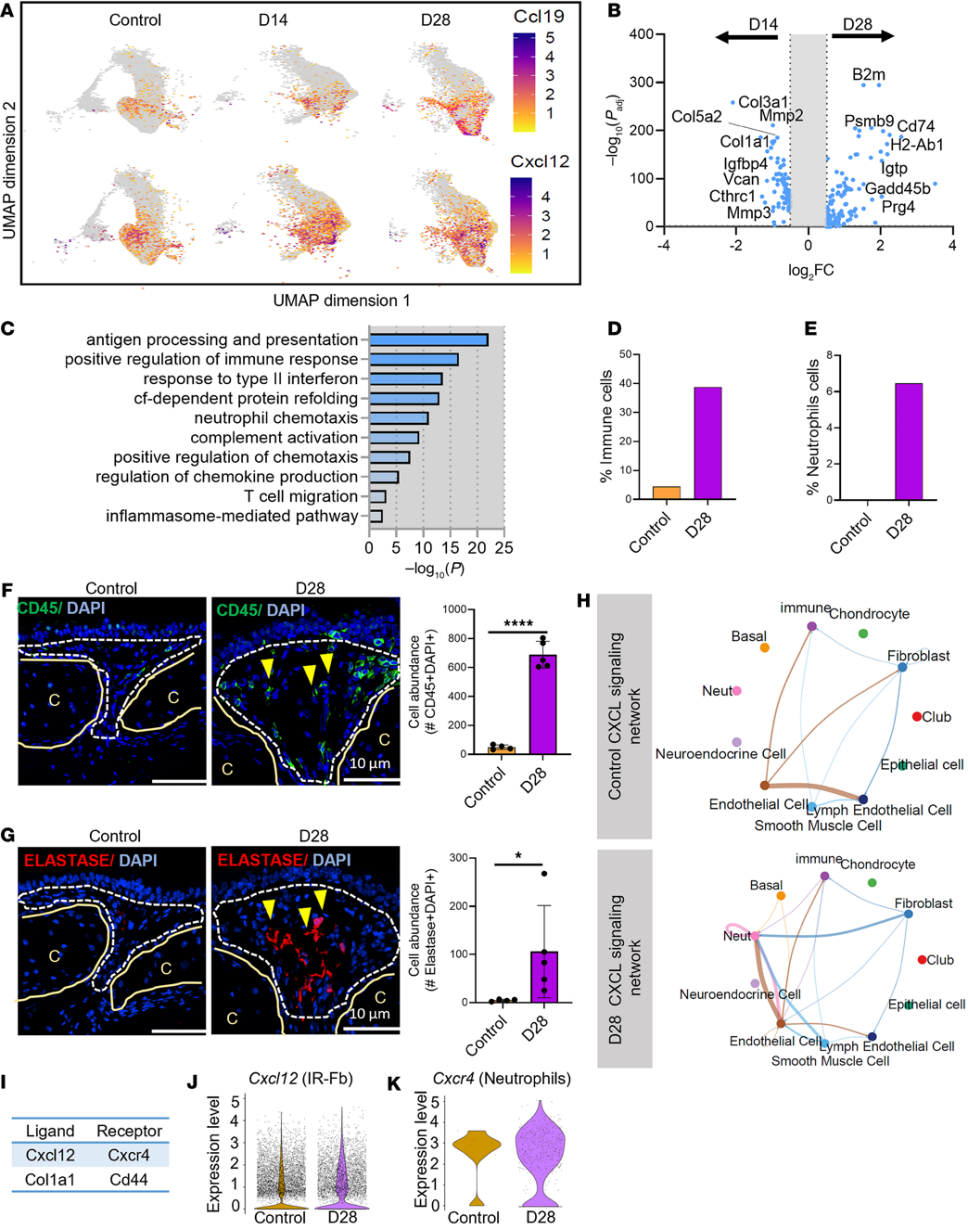
IR-Fb concomitant with immune cell recruitment and infiltration after reconstruction. (**A**) *Ccl19* and *Cxcl12* expression plots on UMAP layout in the normal airway and SIS at D14 and D28. (**B**) Volcano plot of dysregulated expressed genes according to their statistical *P* value (*y* axis) and their relative abundance ratio (log_2_ fold change) between SIS at D28 and D14 identifying immunomodulator-associated genes (*B2m*, *Psmb9*, *Cd74*, *H2-Ab1*, *Cxcl19*, *Prg45*, and *Igtp*). (**C**) GO-enriched pathways corresponding to changes at D28. FDR < 10%. (**D** and **E**) Percentage of immune linages (**D**) and neutrophils (**E**) at normal and D28 after reconstruction obtained from the scRNA-Seq analysis. (**F** and **G**) Representative immunofluorescence staining and corresponding quantification showing the presence of immune cells (CD45^+^, green) (**F**) and neutrophils (ELASTASE^+^, red) (**G**). Yellow arrowheads indicate immune cells expressing CD45. “C” indicates cartilage, and the dotted line indicates the region of interest in the submucosa. (**H**) Circle plots showing the CXCL signaling pathway network between fibroblasts and other airway cell types in the normal airway (upper panel) and in SIS at D28 (lower panel). (**I**) Inferred Fb-immune cell communication ligand-receptor interactions. (**J** and **K**) Violin plots showing *Cxcl12* expression levels in IR-Fb (**J**) and its corresponding receptor *Cxcr4* in neutrophils (**K**). Statistical analysis was performed using unpaired, 2-tailed Student’s *t* test (**F**) and Mann-Whitney test (**G**). *P < 0.05, ****P < 0.0001. Scale bars: 10 µm.

We then focused our attention to the Fb subpopulations that increased in response to SIS. First, we found that immune recruiting Fb subpopulation (characterized by the expression of *Ccl19* and *Cxcl12*) increased in a time-dependent manner (from 1% during homeostasis to 13% and 47% at D14 and D28, respectively) (Figure 3, A and B, and Figure 5A). Evaluation of the IR-Fb DEGs showed an upregulation of ECM components and regulators at D14 and increased expression of immune recruitment factors at D28 (Figure 5B). At the later time point, GO analysis confirmed that this Fb subset exhibited enriched expression of pathways associated with antigen processing and presentation, positive regulation of immune response, and neutrophil chemotaxis (Figure 5C), aligning with an observed increase in immune cells, particularly neutrophils (Figure 5, D and E). Supporting these transcriptomic results, immunofluorescence analysis at D28 after SIS revealed immune cell infiltration (CD45^+^) and neutrophil accumulation (Neutrophil Elastase^+^) within the graft (Figure 5, F and G).

Further elucidation of the interplay between IR-Fb and immune cells using CellChat confirmed communication of IR-Fb with immune cells, especially neutrophils (Figure 5H). Ligand-receptor interaction analysis demonstrated that Fb communication with immune cells was potentially mediated by 2 main pathways, the CXCL pathway, specifically facilitated by *Cxcl12-Cxcr4*, and the Collagen pathway, specifically facilitated by *Col1a1-Cd44* (Figure 5I). Furthermore, increased expression of *Cxcl12* by IR-Fb at D28 was consistent with increased *Cxcr4* expression in neutrophils at the same time point (Figure 5, J and K). Together, our data suggest the increased presence of an IR-Fb subpopulation that plays a modulatory role in immune cell interactions, presumably leading to neutrophil recruitment.

### SIS selectively increases the abundance of Cthrc1^+^ Fb at sites of injury.

**Figure 6:**
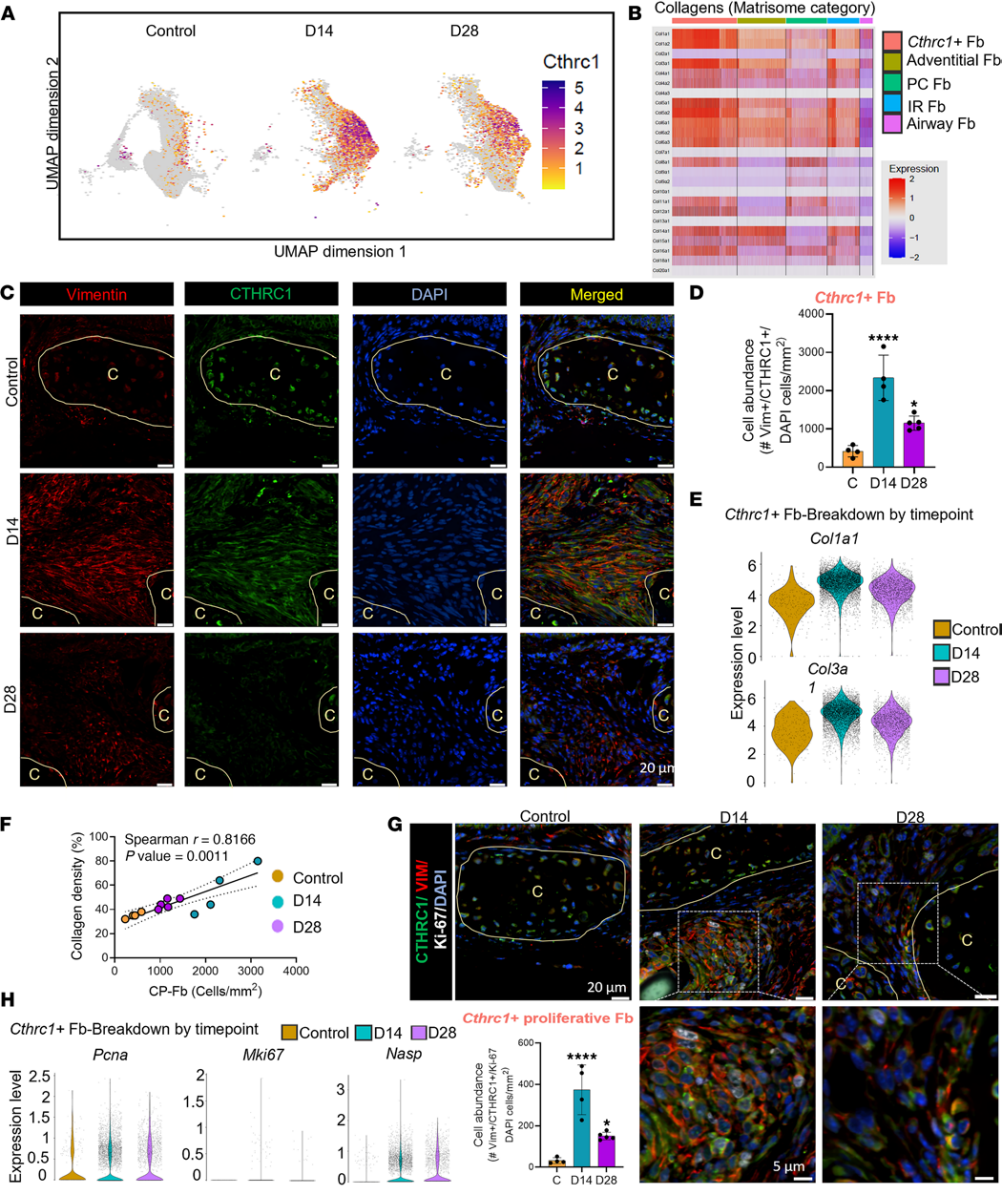
CTHRC1^+^ collagen-producing Fb spatially localize at the sites of injury and has a distinct collagen production. (**A**) *Cthrc1* expression plot on UMAP layout in normal airway and SIS at D14 and D28. (**B**) Heatmap showing the expression levels of genes from Collagens (Matrisome category) in each fibroblast cluster. Red indicates upregulation, and blue indicates downregulation. (**C**) Representative immunofluorescence staining showing the presence of fibroblasts (Vimentin^+^, red) and *Cthrc1^+^* Fb (Vimentin^+^, red; CTHRC1^+^, green). (**D**) Quantification of individual *Cthrc1^+^* Fb per mm^2^ (Vimentin^+^CTHRC1^+^ cells) per condition (Control, *n* = 4; D14, *n* = 4; D28, *n* = 5). (**E**) Violin plots showing higher *Col1a1* and *Col3a1* expression in *Cthrc1^+^* Fb during homeostasis and D14 and D28 after reconstruction. (**F**) Spearman correlation analysis of collagen density and *Cthrc1^+^* Fb numbers. (**G**) Representative immunofluorescence staining showing proliferation (Ki-67, white) of activated *Cthrc1^+^* Fb (Vimentin^+^, red; CTHRC1^+^, green) and quantification of individual proliferative *Cthrc1^+^* Fb per mm^2^ (Vimentin^+^CTHRC1^+^Ki-67^+^ cells) per condition (Control, *n* = 4; D14, *n* = 4; D28, *n* = 5). (**H**) Violin plots showing higher *Pcna*, *Mki67*, and *Nasp* expression in *Cthrc1^+^* Fb during homeostasis and D14 and D28 after reconstruction. Data are shown as mean ± SD. Statistical analysis was performed using 1-way ANOVA with Dunnett’s multiple-comparison test. ***P <0. 001. Scale bars: 5 µm and 20 m as indicated.

*Cthrc1^+^* Fb were the most abundant subtype in response to SIS and were likely to be differentiating from Adventitial-Fb (Figure 3, A and B). Following a similar pattern of their abundance, we confirmed that *Cthrc1* expression in pseudotime was mainly restricted to this subpopulation and was higher at D14 compared with other time points (Figure 6A). Consistent with this transcriptomic signature, immunostaining confirmed the presence of activated Fb at D14 after SIS using CTHRC1 as well as additional markers, COL1 and Periostin (Figure 2B and Supplemental Figure 6A).

Consistent with previous studies where CTHRC1 expression in Fb has been associated with an activated phenotype characterized by increased collagen production (26), *Cthrc1^+^* Fb, among all identified Fb subtypes, showed the highest expression levels of collagen genes (matrisome category) (32). Specifically, we found that the main fibrotic-associated collagens *Col1a1* and *Col3a1* were the most upregulated (Figure 6B and Supplemental Table 2).

To further evaluate the spatiotemporal emergence of *Cthrc1^+^* Fb in the reconstructed airway, we employed a multiplex immunofluorescence approach. As expected, we found very few *Cthrc1^+^* Fb during homeostasis. However, SIS transiently increased their abundance (Figure 6, C and D, and Supplemental Figure 6B). Spatially, *Cthrc1^+^* Fb were found in the sites of injury in submucosal, paratracheal, and anastomotic regions, surrounded by collagen fibers (Figure 6C and Supplemental Figure 6, A and C).

Considering that *Cthrc1^+^* Fb have been implicated in regulating pathologic scarring in fibrotic conditions (26, 30), we next examined the proximity of this population with respect to dense collagen areas. Regionally, *Cthrc1*^+^ Fb correlated with collagen deposition after reconstruction (Supplemental Figure 6C). To further understand the connection between high collagen deposition and *Cthrc1^+^* Fb abundance, we performed a correlation analysis. Using this strategy, we showed that the levels of collagen deposition coincided with the abundance of *Cthrc1^+^* Fb and was independent of SIS (Figure 6, E and F). To further characterize if SIS also promotes shifts in *Cthrc1^+^* Fb collagen expression, we measured the level of expression of *Col1a1* and *Col3a1* in homeostasis and postsurgical reconstruction at D14 and at D28 in the *Cthrc1^+^* Fb. Mirroring the fibrosis trend, *Cthrc1^+^* Fb–mediated expression of *Col1a1* and *Col3a1* was transiently increased at early time points (Figure 6E). Furthermore, GO analysis of the biological function of *Cthrc1^+^* Fb DEGs revealed enrichment of pathways associated with ECM remodeling, respiratory tube development, and glucose homeostasis (Supplemental Figure 6D). Notably, potential transcription regulators included *Jun* and *Smad3*, downstream factors of the fibrotic master regulator TGF-β (Supplemental Figure 6E).

Beyond differentiation from Adventitial-Fb, we then explored the contribution of proliferation on *Cthrc1^+^* Fb abundance (Figure 6, G and H). SIS increased Ki-67 expression in *Cthrc1^+^* Fb, histologically (Figure 6G). Transcriptomic analysis revealed that *Cthrc1^+^* Fb exhibited elevated expression of proliferation-associated genes (*Pcna*, *Mki67*, and *Nasp*) (Figure 6H), thus confirming that cell proliferation significantly contributes to the expansion of this subpopulation. Together, our findings indicate that SIS leads to the transient emergence of a unique collagen-producing Fb subpopulation that accumulates at the injury site.

### Fb-airway basal cell crosstalk via TGF-β and ncWNT modulates activation.

To identify potential cellular interactions that regulate Fb transitional states and activation, we used the CellChat package (33). SIS response showed a significant increase in the number (Supplemental Figure 6F) and strength (Supplemental Figure 6G) of inferred interactions compared with homeostasis. We then visualized the crosstalk of inferred interactions between Fb and other airway cell types in response to SIS using chord plots (Supplemental Figure 6H). Among all cell types, Fb showed the highest autocrine and paracrine signaling communications in response to SIS (Supplemental Figure 6H)

**Figure 7:**
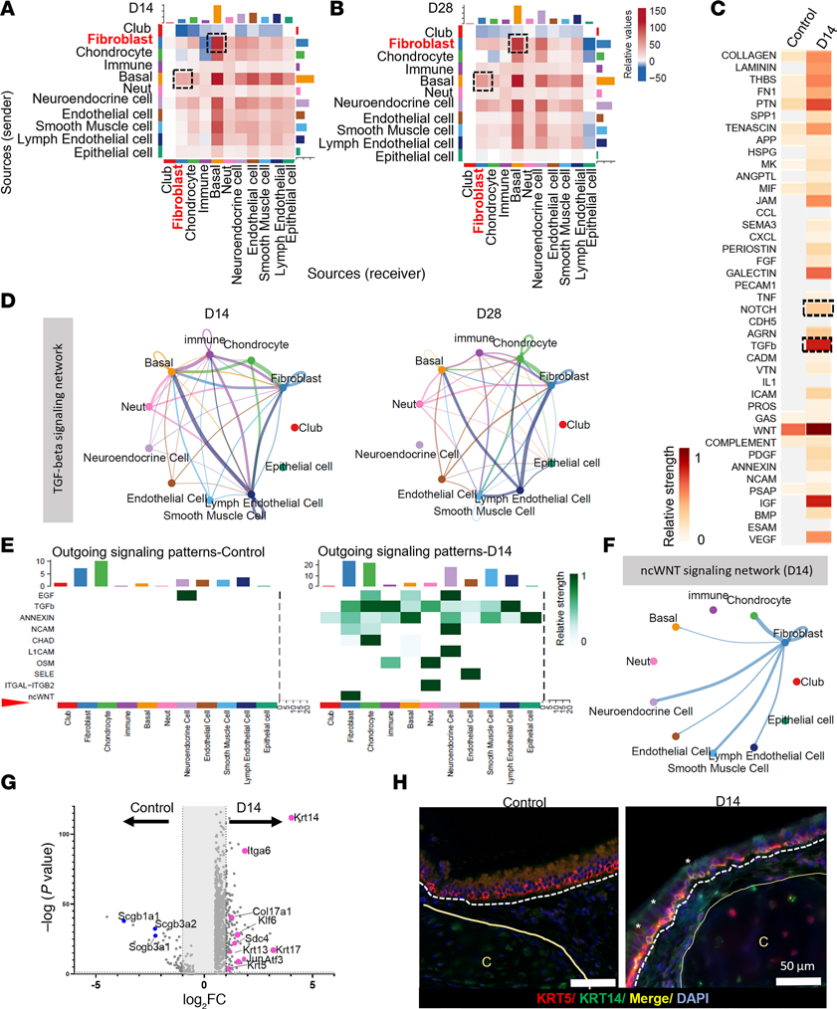
Fibroblast-basal cell communication mediated by TGF-β. (**A** and **B**) Heatmap of the inferred interaction between sender and receiver in SIS at D14 (**A**) and D28 (**B**) versus Control. (**C**) Heatmap of top inferred pathways. Stemness pathways (TGF-β, NOTCH, WNT, and FGF) activated in the basal cell cluster in response to SIS at D14 are highlighted in a dotted box. (**D**) Circle plots showing the TGF-β network between fibroblasts and other airway cell types in response to SIS at D14 (left) and D28 (right). (**E**) Heatmap of the outgoing signals driven by all identified airway cell types during homeostasis or at early repair time points (D14). Green intensity indicates strength of the signal. (**F**) Circle plots showing the ncWNT network between fibroblasts and other airway cell types in response to SIS at D14. (**G**) Volcano plot of dysregulated expressed genes according to their statistical *P* value (*y* axis) and their relative abundance ratio (log_2_ fold change) between normal airway and SIS at D14 identifying stemness-associated genes (*Krt14*, *Itga6*, *Krt17*, *Col17a1*, *Klf6*, *Sdc4*, *Krt13*, *Krt5*, *Jun*, and *Atf3*). (**H**) Representative immunofluorescence staining basal cells (KRT5) or activated basal cells (KRT5/KRT14–double-positive cells) in the homeostatic airway (left panel) or reconstructed airway at D14 (right panel). White asterisks indicate double-positive cells, and the dotted line indicates the epithelium from the submucosa. Scale bars: 50 µm.

Subsequent analysis using sender-receiver interaction revealed that Fb preferentially communicated with airway basal epithelial cells in a bidirectional manner in response to SIS (Figure 7, A and B). To further explore the basis of this communication, we evaluated activated signaling pathways in airway basal cells and found that Notch, TGF-β, and WNT pathways were more active in basal cells responding to SIS compared with during homeostasis (Figure 7C), and we found that TGF-β signaling had the highest relative strength when compared with control levels. Remarkably, during homeostasis, we could not identify any inferred communication via the TGF-β signaling pathway among the different airway cell types. However, in response to SIS, TGF-β–dependent communication increased, and Fb were receiving both autocrine and basal cell–mediated paracrine signals facilitated by this pathway, especially at the early repair time point (Figure 7D). Notably, Fb also sent paracrine TGF-β signals to the basal cells, confirming a bidirectional intercellular communication.

To explore additional potential pathways that could be participating in Fb-basal cell communication, we determined the outgoing signals sent by Fb (Figure 7E). We found that noncanonical WNT (ncWNT) was a signal exclusively driven by Fb at early but not late time points and that they operated in both an autocrine and paracrine manner (Figure 7F). In the airway, it has been previously reported that initiation of ncWNT signaling is necessary during injury/repair and that impaired initiation results in inadequate upregulation of basal cells activation programs (34). Previously, we found that airway basal cells are activated and respond to injury following airway reconstruction (22). In alignment with those findings, examination of the DEG profile of airway basal cells showed upregulation of genes related to basal cell activation including *Krt14*, *Krt13*, *Krt17*, *Krt5*, *Col17a1*, and *Klf6* (Figure 7G) and the ncWNT key downstream transcription factor, Jun (Figure 7G). Furthermore, immunostaining for activated basal cells (KRT5/KRT14) revealed their presence exclusively in response to SIS (Figure 7H).

Given that previous evidence has shown that TGF-β can induce CTHRC1-dependent Fb activation during tissue repair (26), we hypothesize that, similarly in SIS, TGF-β signaling activation contributes to this phenomenon. Consistent with this idea, we found that the 3 TGF-β receptors (*Tgfbr1*, *Tgfbr2*, and *Tgfbr3*) were expressed in the *Cthrc1^+^* Fb at all time points; however, *Cthrc1^+^* Fb preferentially expressed *Tgfbr2* in response to SIS (Supplemental Figure 7A). Additionally, we observed a similar pattern of expression when we explored canonical TGF-β downstream genes: *Smad3*, *Smad4*, and *Fn1* in *Cthrc1^+^* Fb in response to SIS, confirming activation of this signaling in *Cthrc1^+^* Fb (Supplemental Figure 7B). Collectively, these findings suggest that SIS is characterized by increased cellular interactions promoting mutual activation of Fb and basal cells, potentially mediated by the TGF-β pathway.

### TGF-β induces CTHRC1^+^ Fb activation and modulates collagen production in human Airway-Fb.

To identify clinical translatability of our findings, we conducted transcriptomic analysis in the human tracheobronchial airway using the Tabula Sapiens data set (35) (Supplemental Figure 7, C and D, and Supplemental Figure 8, A–E). We identified human Fb subpopulations that exhibited similar transcriptomic profiles to the 5 identified mouse Fb subpopulations (10 gene markers set of each mouse Fb subset). Using this strategy, we confirmed the presence of cells expressing selected gene sets corresponding to Adventitial-Fb (Supplemental Figure 8A), Airway-Fb (Supplemental Figure 8B), PC-Fb (Supplemental Figure 8C), *CTHRC1^+^* Fb (Supplemental Figure 7D), and IR-FB (Supplemental Figure 8D). Furthermore, histogram representation of each set mean intensity confirmed the variation of the expressed gene sets among the total Fb population (Supplemental Figure 8E).

After we identified the presence of *CTHRC1^+^* Fb in the human airway, we stained CTHRC1 in pediatric tracheobronchial airway tissue from resected specimens obtained during airway reconstruction surgeries. In parallel with the preclinical model, where *Cthrc1^+^* Fb were localized within fibrotic regions (Supplemental Figure 6, A and B), *CTHRC1^+^* cells were similarly distributed within fibrotic areas in the human reconstructed airway (Supplemental Figure 8, F–H).

To further corroborate the effect of TGF-β signaling in modulating *CTHRC1*^+^ activation and collagen production in Fb in response to SIS, we isolated primary human tracheal Fb (HTrFb) from pediatric tracheobronchial airway tissue from resected specimens obtained during airway reconstruction surgeries and treated them with TGF-β. Since resected samples vary in size and are obtained from different areas, we evaluated the heterogeneity of our cultures by immunostaining for PI16 and quantifying positive and negative cells. We found that the frequency of Adventitial-Fb in each culture varied from 31% to 53% prior stimulation and that TGF-β treatment reduced this proportion to 16%–33% (Supplemental Figure 7, E and F). In contrast, we found that TGF-β increased the frequency of *CTHRC1^+^* Fb, while vehicle-treated Fb expressed scarce levels of CTHRC1 (Supplemental Figure 7, G and H). To confirm the critical role of TGF-β in CTHRC1^+^ Fb activation and collagen production, we abrogated TGF-β signaling and evaluated Fb activation (CTHRC1^+^) and collagen synthesis (COL1). We found that TGF-β treatment not only induced CTHRC1^+^ activation and collagen production but promoted the formation of focus-like structures. Abrogation of TGF-β signaling by SB431542 was sufficient to decrease CTHRC1 and COL1 protein expression in Airway-Fb to vehicle levels and inhibit the formation of focus-like structures (Supplemental Figure 7, I–K). Overall, our findings indicate that Fb become activated during SIS, presumably through the TGF-β pathway, thereby promoting excessive collagen production.

### Repeated SIS favors the emergence of activated CTHRC1^+^ Fb, affecting Fb-basal cell crosstalk.

**Figure 8:**
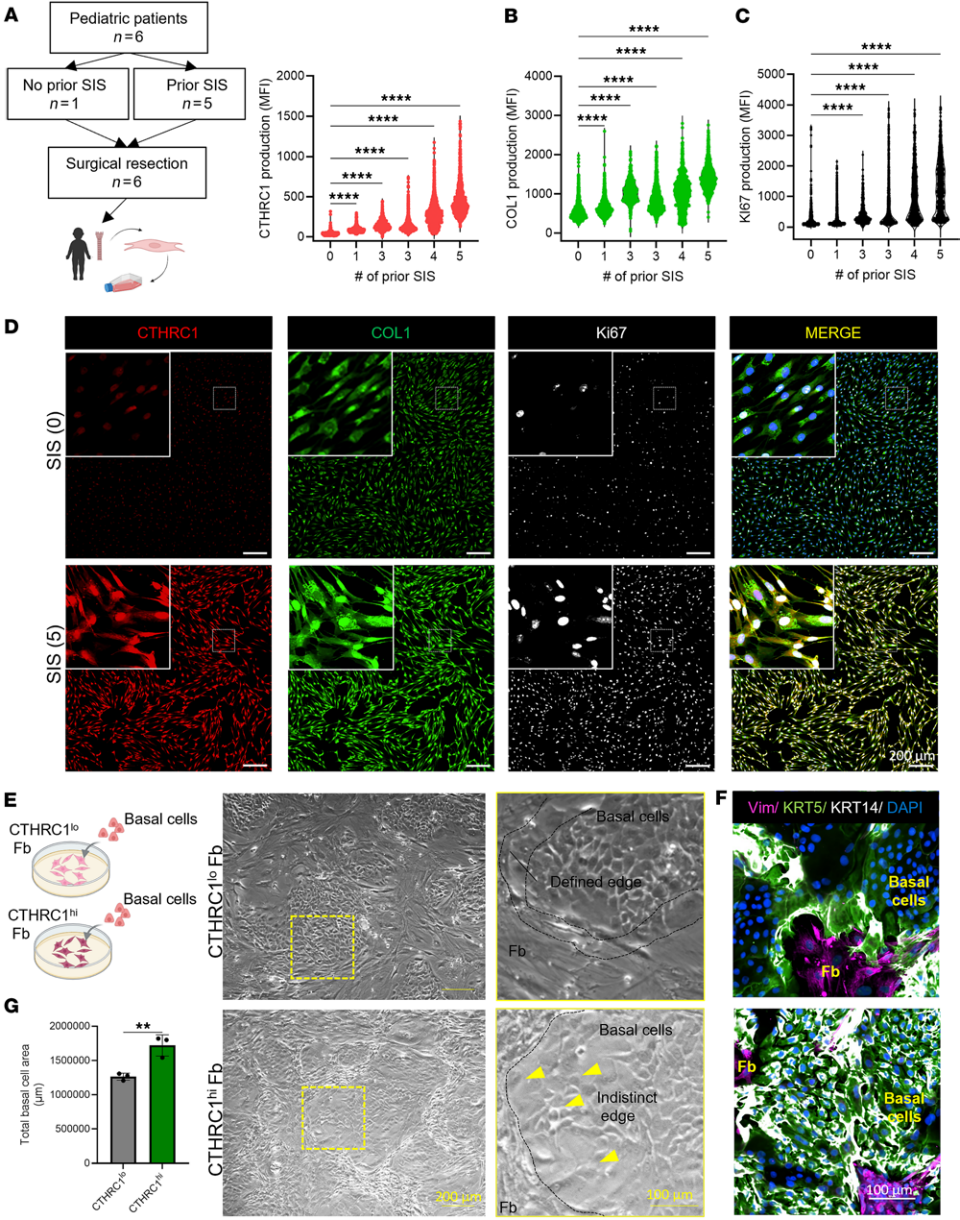
Repeated SIS in the airway leads to CTHRC1^+^ Fb activation and affects fibroblasts-basal cell crosstalk. (**A**–**C**) Schematic of surgical resection and quantification of the fluorescence intensity within the boundaries of a single cell of CTHRC1 (**A**), COL1 (**B**), and Ki-67 (**C**) in fibroblasts isolated from patients that were or were not subjected to SIS before sample collection (*x* axis represents number of prior SIS of a particular patient, *n* = 6). (**D**) Representative immunofluorescence staining of CTHRC1, COL1, and Ki-67 on human isolated fibroblast from patients with different surgical clinical history. (**E**) Schematic of basal cell collection and representative light microscope images of images of cocultures containing human basal cells and an irradiated fibroblast feeder layer composed of cells expressing low or high levels of CTHRC1 (CTHRC1^lo^ Fb, upper panels; CTHRC1^hi^ Fb, bottom panels). (**F**) Representative immunofluorescence staining of basal cell activation markers KRT5 and KRT14 and Vimentin in cocultures. (**G**) Quantification of total basal cell area after 5 days of coculture on fibroblasts expressing different levels of CTHRC1. Statistical analysis was performed using Kruskal-Wallis test (**A**–**C**) and unpaired, 2-tailed Student’s *t* test (**G**). **P < 0.01, ****P < 0.0001. Scale bars: 100 µm and 200 µm as indicated.

To further address the clinical significance of SIS on Fb activation, we evaluated the expression of CTHRC1 in Fb isolated from airway tissue resected from pediatric patients who were or were not subjected to SIS and categorized them based on their surgical history. Our findings revealed that levels of CTHRC1 correlate directly with the number of airway surgical interventions (prior SIS) performed on the airway before sample collection (Figure 8, A and D). Furthermore, human *CTHRC1^+^* Fb increased both collagen production and proliferation in response to SIS, mirroring the mouse model findings. (Figure 8, B–D). Since our cell-to-cell communication analysis identified Fb-basal communication after SIS, we wanted to determine if differentiation into the CTHRC1^+^ activation state would have an effect on basal cell function. To address this question, we performed a functional analysis using our previously described monolayer coculture model (36, 37), in which Fb with low (CTHRC1^lo^ Fb) or high (CTHRC1^hi^ Fb) CTHRC1 expression levels were used as irradiated feeders (Figure 8, E–G). Primary human basal cells formed well-defined colonies among normal Fb feeders (37). Similarly, when human basal cells were cocultured with CTHRC1^lo^ Fb, they formed colonies with a defined edge of KRT5/KRT14–double positive cells (Figure 8, E and F). In contrast, coculture with CTHRC1^hi^ Fb affected basal cell colony morphology, resulting in irregular and larger colonies of KRT5/KRT14–double positive cells (Figure 8, E–G). Collectively, our findings indicated that differentiation into a CTHRC1^+^ Fb, enhanced by repeated SIS, influenced basal cell activation, thereby promoting KRT5/KRT14 expression and increased surface area, likely by stimulating basal cell proliferation.

## Discussion

Fb are a heterogeneous population with “universal” and tissue-specific transcriptional programs, which are conserved across mice and humans (26). Despite the relevance of Fb heterogeneity and their diverse functions in scarless and fibrotic repair in the airway, these aspects have not been well described in the setting of anastomotic surgical injury. Our transcriptomic study presents a map of the Fb subpopulations and indicates the sequence of events of their orchestrated contribution to tissue repair. While there is evidence of Fb plasticity and a role for this process in tissue regeneration and repair; specific injuries/insults can modulate the transcriptional programs that define their capacity to acquire different activation states and subsequently implement repair responses (26, 31). Here, we identify that progression through the repair process from SIS involves coordinated changes in the abundance of Fb subpopulations and selective emergence of transcriptionally heterogeneous activated states. The significance of this finding is supported by: (a) the identification of a similar subpopulation (*Cthrc1^+^* Fb) that was previously described in human pulmonary fibrosis and injured lungs, (b) the presence of an immunomodulatory subpopulation that is likely to be coordinating neutrophil infiltration, and (c) the unexpected detection of a subpopulation of fibrocartilage-like structures of cells that are molecularly and structurally similar to both Fb and chondrocytes.

Fb differences across organs have largely been attributed to lineage origin and tissue regeneration capacity (38). In organs such as the lungs where regeneration occurs secondary to an injury, Fb primarily contribute to ECM turnover during homeostasis (26, 29). Similarly, in the airway we identified that native airway and Adventitial-Fb are resting subpopulations that, in response to injury, differentiated into activated states and contributed to the SIS-induced Fb profile.

*Cthrc1^+^* Fb have traditionally been considered a pathologic collagen-producing subpopulation that emerges in response to bleomycin injury and is overrepresented in human pulmonary fibrosis (26). It is assumed that this subtype appears only in the fibrotic lung and is responsive to the profibrotic milieu. However, the *Cthrc1^+^* Fb population was transiently present after SIS, which does not involve an external profibrotic stimulus, suggesting a change in this paradigm. Importantly, the observed *Cthrc1^+^* Fb subpopulation could be different from the one previously reported in the bleomycin model since they emerged from distinct Fb subpopulations (26, 27). Therefore, future directions will compare the similarities and differences of their transcriptomic signatures and focus on identifying the specific role of CTHRC1 in airway repair.

The appearance of the *Cthrc1^+^* Fb subpopulation has also been associated with epithelial damage, where the loss of epithelial cells alone is sufficient to induce transitional state programs (39) that precede the accumulation of *Cthrc1^+^* Fb (29). Conversely, in our SIS model, epithelial cell loss does not occur. Instead, the process involves a molecular activation of differentiation programs, suggesting that alternative mechanisms contribute to the emergence of *Cthrc1^+^* Fb in the airway. *Cthrc1^+^* Fb have previously been reported to influence epithelial progenitor AT2 cell homeostasis in the lung (40). In our study, we demonstrated that CTHRC1^+^ Fb likely affect the basal cell microenvironment by inducing activation programs in tracheal basal cells.

Our findings indicate that this communication is mediated by 2 signaling pathways: ncWNT and TGF-β. Fb predominantly transmit ncWNT and TGF-β signals to basal cells, while basal cells exclusively send TGF-β signals to Fb. ncWNT plays a critical role in regulating airway basal cell proliferation (34); here, we observed that SIS-activated CTHRC1^+^ Fb promoted basal cell expansion. Although our results do not directly identify the specific ncWNT mediators involved in this communication, we observed the involvement of the downstream transcription factor *Jun*, which is activated via JNK. Jun has been associated with processes such as cell migration, polarity, and cytoskeletal rearrangement (41). Further studies will be needed to explore the exact role of ncWNT in airway basal cell expansion.

Our analysis further emphasized the predominant role of TGF-β as a key inductor to the *Cthrc1^+^* activated state, and understanding TGF-β–mediated crosstalk between the epithelium and Fb may enable strategies that are designed to modulate remodeling and repair. Previous studies in the heart have revealed that the deletion of *Cthrc1* resulted in ventricular rupture and significant loss of the “collagen scaffold” that is necessary for proper repair after myocardial infarction (17). This study and ours suggested that tissue injury is accompanied by shared repair mechanisms that involves the emergence of *Cthrc1^+^* Fb, and therapeutic strategies should be focused on regulating their accumulation and collagen production rather than inhibiting their emergence.

In addition to their plasticity and heterogeneity, Fb are known to communicate with other cell types, including immune cells (19). Neutrophils are the primary responders during repair and can promote healing or activate pathways that lead to inflammation and fibrogenesis (42). In our previous study, we revealed a major role of neutrophils and their products during late repair in response to SIS (23). In this study, *Ccl19^+^* Fb, a rare subpopulation found during homeostasis, significantly increased their abundance in response to SIS. CellChat analysis predicted a paracrine communication between this subpopulation and immune cells, especially neutrophils. Future work will focus on evaluating the mechanisms behind this communication.

At early time points, IR-Fb expressed high levels of matrix metalloproteinases (MMPs), including MMP2. MMP2 (also known as gelatinase A) cleaves and activates CXCL12 and processes S100 protein (43), contributing to the creation of a chemotactic gradient and subsequent immune cell recruitment at late time points. While additional studies are needed, these findings suggest that Fb may orchestrate the previously observed changes in the immune milieu in response to SIS.

A unique aspect of our model is identification of fibrocartilage-like structures. We followed these structures for a year and documented their similarity with the tracheal cartilage. Furthermore, we showed that these structures increased in size and frequency upon injury, and we suggest that they may augment the mechanical properties of the acutely damaged cartilage. Our scRNA-Seq and immunofluorescence studies uncovered an unexpected lineage relationship between perichondrial Fb and chondrocytes. This function was previously presented (31) but has not been validated and will require the generation of specific genetic lineage tracing tools to experimentally verify the computational predictions. If so, there are significant clinical implications for this process. Previous studies have highlighted the medical relevance of mesenchymal cells, such as Fb, in bioengineered and decellularized scaffolds for tracheal replacement (43). However, these strategies have limitations, including a lack of understanding regarding the mechanisms involved in vivo. Therefore, understanding the differentiation capacity of perichondrial Fb is crucial for advancing tracheal regeneration.

We acknowledge that our study has limitations. The preclinical microsurgical murine model was performed in healthy mice and does not recapitulate all aspects of human airway surgery such as preexisting conditions. Our transcriptomic analysis evaluated the early and late stages of repair, but this process remains to be determined at longer time points. In this study, we did not determine if cartilage damage alone is sufficient to trigger Fb-chondrocyte differentiation, and future work using a cell depletion model will be required to answer this question. Despite these limitations, the role of distinct Fb subpopulations during anastomotic repair warrants further investigation for the development of therapies.

Our study demonstrates that ideal outcomes following tracheobronchial surgery involve a highly orchestrated response from Fb subsets. Surgical complications in airway reconstruction bear the hallmarks of an imbalance of this response, manifesting as either anastomotic leak (insufficient repair) or stenosis (overabundant fibrosis and repair). To develop therapeutic strategies that decrease airway complications after reconstruction (stenosis/fibrosis), preclinical studies have focused on drugs that reduce inflammation and fibrosis of the trachea after tracheotomy, inhibit proliferation and transformation of Fb and secretion of ECM, as well as inhibit TGF-β1 in the scar tissue. However, clinical use of antiinflammatory medications has shown inconsistent results, and there remains a lack of targeted therapy that serves as an adjunct to optimize surgical outcomes. Our study offers potentially new insights into Fb biology and the heterogeneity of the Fb that contribute to fibrosis and stenosis following tracheobronchial surgery. An expanded understanding of Fb dynamics, interactions with the immune system and inflammatory mediators, and specific repair roles has the potential to foster the development of targeted therapeutics that could curtail the aberrant repair states and improve outcomes after airway reconstruction.

## Methods

### Sex as a biological variable.

TrFb were cultured from tracheal tissue obtained from direct laryngoscopy with bronchoscopy derived patients of both female and male sexes that required tracheal resection. The detailed description of patient demographics and clinical data can be found in Supplemental Table 1. Female mice were utilized in mouse microsurgical experiments. Sex was not evaluated as a biological variable in the experiments.

### Study design.

In this study, our objective was to investigate how SIS alters Fb response and orchestrates repair to airway reconstruction using a preclinical microsurgical model of segmental tracheal transplantation that mimics human end-to-end tracheobronchial surgery. The in vivo mouse airway reconstruction model was established by transplanting a 5 mm segment of trachea collected from below the third ring to a genetically identical host. Characterization of airway dimensions and calcification was performed by μ-CT. Tracheal tissue was harvested at D14 and D28 for all experiments except those mentioned otherwise. Macroscopic changes to the submucosa and fibrosis were measured by histology. scRNA-Seq analysis was performed to investigate molecular and cellular changes. The findings were validated using both quantitative and qualitative analysis. All number of biological replicates and statistics are listed in the figure legends and data analysis. Sample size was not determined using a statistical method. Animals assigned to different groups were randomized. μ-CT analysis was blinded. Additional mechanistic studies of cellular activation were conducted in vitro using primary cells isolated from patients who underwent airway reconstruction.

### Human tracheal tissue.

Human tracheal tissue was obtained from direct laryngoscopy with bronchoscopy for tracheal resection from the site of injury (Supplemental Table 1).

### Airway reconstruction model and postoperative care.

To establish the in vivo model, a 5 mm segment of the trachea was excised from below the third tracheal ring and transplanted immediately into a genetically identical recipient, as previously described (25). Animals were provided buprenorphine (0.03 mg/kg, s.c.), placed in a recovery cage on a heating pad until ambulatory, and provided ibuprofen (30 mg/kg) for 48 hours in drinking water. Daily observation for signs of respiratory distress, poor grooming, or weight loss was conducted. Unresolved respiratory distress or weight loss > 20% were criteria for early euthanasia.

### Tissue harvesting.

Host mice were sacrificed for organ harvest at established time points after reconstruction by an i.p. overdose of ketamine/xylazine cocktail (200 mg/kg ketamine, 20 mg/kg xylazine, 10 mg/kg ketoprofen). Grafts were harvested following euthanasia and processed accordingly.

### Histological quantification.

Submucosal thickness was measured as described previously (25). Collagen content was measured using sections stained with Masson’s trichrome and the ImageJ (NIH) Color Thresholding tool. Collagen content was defined as the percentage of the total blue area of the submucosa (hue: 120–168; saturation: 15–255; brightness: 0–255). Collagen content measurements were taken within the midgraft of the reconstructed area. For consistency, measurements for the native trachea were limited to 2 centermost cartilages of the longitudinal section.

### Histological quantification of the fibrocartilage-like structures.

To calculate the frequency in which we found a fibrocartilage-like structure present in a cartilage ring, we measure their frequency as described in this formula:


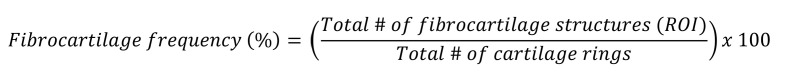


Additionally, we measured the percentage of the area that corresponds to the fibrocartilage-structure in terms of the total area (fibrocartilage-structure + cartilage ring). As described in this formula:
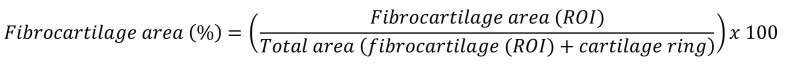


### Immunofluorescence quantification.

Tissue sections were deparaffinized, rehydrated, subjected to antigen retrieval, and processed as previously described (44) with small modifications. Briefly, after blocking with 10% donkey serum in PBS for 1 hour at 4°C, tissue sections were incubated overnight at 4°C with primary antibodies against chicken anti-vimentin (NB300-223; lot 6815/120522; 1:75; Novus Biologicals), rabbit anti-CTHRC1 (16534-1-AP; lot 00124580; 1:75; Proteintech), rat/anti–mouse Ki-67 (Ref 14-5698-82; lot 2747806; 1:200; Invitrogen), rabbit anti-CD45 (ab10558; lot GR308463-1; 1:100; Abcam), SOX9 (AB5535MI; lot 4018162; 1:400; MilliporeSigma), COL1 (Ref MA1-26771; lot YA3811471; 1:200; Invitrogen), rabbit anti–neutrophil elastase (PA5-115648; lot ZJ4499836A; 1:200; Invitrogen), rabbit anti-KRT5 (905501;lot B374080; 1:500; BioLegend), mouse anti-KRT14 (MA5-115999; lot YB372387; 1:500; Invitrogen), goat anti-hPI16 (AF4980; lot CBEB0224011; 1:200; R&D), rat anti-periostin (MAB3548; lot YCF0422021; 1:200; R&D), or COL2 (NBP177795; lot 43393; 1:200; Novus Biologicals). The next day, sections were incubated with secondary antibodies as follows: Alexa Fluor 647 donkey anti–chicken IgY (A32933; lot XI355385; 1:200; Invitrogen), Alexa Fluor 488 donkey anti–rabbit IgG (A21206; lot 2541645; 1:200; Invitrogen), Alexa Fluor 568 donkey anti–rat IgG (A78946; lot 2570534;1:500; Invitrogen), Alexa Fluor 488 DAM donkey anti-mouse (R377114; lot 2845304;1:500; Invitrogen), Alexa Fluor 546 donkey anti–goat IgG (A11056; lot 2566344;1:500; Invitrogen), and Alexa Fluor 594 donkey anti-rabbit (A21207; lot 2066086;1:500; Invitrogen) for 1 hour at room temperature. Following this, tissue sections were treated with TrueVIEW autofluorescence quenching reagent (Vector Laboratories, SP-8400-15) for 2 minutes and mounted using ProLong Gold Antifade Mountant with DAPI following manufacturer’s instructions (Thermo Fisher Scientific). Images were captured with Nikon AX R confocal on a Ti2-E base using Nikon Plan Apo Lambda D in 20x (0.8 NA) objective or on a Zeiss Axio Imager.M2 using Zeiss Plan Apochromat in 20× (0.8 NA) objective.

For mouse airway tissue, multichannel images of longitudinal sections were captured as *Z*-stacks using NIS-Elements AR (Nikon Instruments, v 5.42, 20× Nikon Plan Apo Lambda D objective with 0.8 NA), and *Z*-stacks were compressed into 2D images using the Extended Depth of Focus (EDF) module in NIS-Elements. Quantification was done by counting positive cells expressing (vimentin, vimentin^+^CTHRC1^+^, vimentin^+^CTHRC1^+^Ki-67^+^, vimentin^+^POSTN^+^, COL1^+^, CD45^+^, or neutrophil-elastase^+^) using the Cell Counter plug-in of ImageJ software (NIH) as previously reported (22). All fields were evaluated for quality by a single reviewer. The area of each region of interest was measured in square millimeters to calculate total cellularity (cells/mm^2^).

Images of Fb cultures and cocultures with basal cells were batch analyzed identically using NIS-Elements (v 5.42), and custom automated analysis algorithms were created in the General Analysis 3 (GA3) software module.

Fb multichannel images were denoised to assist automated segmentation using the denoise.ai tool in the NIS.ai software module, and a large radius (50 μm) rolling ball step was used to subtract any background in all channels. Nuclei were selected by automated thresholding in the DAPI channel, and a low fixed threshold in the COL1 channel was used to segment all cell pixels from the background. A watershed function was then used to separately segment the total cell area belonging to each nucleus based on all 4 channels. The mean COL1 and CTHRC1 signal intensity was measured for each entire cell, and the mean signal intensity of Ki-67 was measured for each nucleus. The data were reported for each cell individually and as a mean for all cells in each image.

Fb and basal cell coculture multichannel *Z*-stacks were compressed into 2D using the EDF software module, and the EDF images were denoised using the denoise.ai tool in the NIS.ai software module. A 15 μm radius rolling ball step was used to subtract any background in the DAPI and Vimentin channels. Nuclei were segmented by automated thresholding in the DAPI channel, Fb were segmented using a fixed threshold in the vimentin channel, and basal cells were segmented as the overlap of the KRT5 and KRT14 signal. Nuclei counts belonging to Fb or to basal cells based on overlap with Vimentin, and the number, mean size, and the total area of basal cell colonies in each image were measured and reported.

### scRNA-Seq.

scRNA-Seq data from our previously published dataset (deposited to NCBI-SRA with the accession number PRJNA954770) (22) on native mouse trachea and tracheas at 14 and 28 days after SIS, and the human tracheobronchial airway data set available on the Human Cell Atlas (as defined by the authors) (35) was obtained. Single-cell analysis was performed as previously described (22). Briefly, an average of 10,000 cells per sample were processed on the Chromium Next GEM Single Cell 3′ kit at the Genomics Shared Resource at the Ohio State University Comprehensive Cancer Center and sequenced with Novaseq at the Institute of Genomic Medicine at Nationwide Children’s Hospital. Single-cell reads were demultiplexed and aligned to the mouse genome reference (ENSEMBL, mm8) using Cell Ranger (v.5.0.1, 10x Genomics). Quality control and filtering were performed to remove cells expressing fewer than 200 genes, doublets, and apoptotic cells. Only genes detected in at least 3 cells were included. The counts were integrated separately, normalized, and scaled, and highly variable genes were identified for a principal component analysis to determine the dimensionality of cells using the Seurat Package (version 4.0) in R. After normalization, we detected and removed doublets and applied ambient RNA correction prior to clustering. Cell clusters were identified using the Louvain method and visualized with UMAP. A model-based analysis of single-cell transcriptomics (MAST) framework was used to identify differentially expressed genes from each cluster. Differentially expressed genes from each cluster were used to annotate the clusters (31, 45–47) and manual annotation. Fb and immune cells were then subsetted out from the datasets to better define heterogeneity before combining them into a UMAP for analysis. Subclusters were defined using FindAllMarkers and named them using previously documented marker genes (26, 31). For enrichment analysis, DEPs were analyzed. Among the commonly used enrichment analysis methods, we used grouping function from Metascape (48) to filter out results with high similarity, applying default parameters as previously reported (23).

### Trajectory analysis.

Trajectory inference on the Fb and chondrocyte subset from Control, D14, and D28 was performed using Monocle3 (package version 4.0.3) as previously described (22). In brief, we first determined the landmark cells or the starting point of the trajectory by employing the get_earliest_principal_node function using its default parameters, which objectively determined the root cells based on the trajectory structure. After defining “landmark” cells, we generated a principal graph that defined the trajectories. The principal graph was used as a guide to contract a graph on all the cells where the pseudotime of each cell could then be computed back to the assigned root node. Plots were generated within Seurat and Monocle3 or using the ggplot2 package.

### Human Fb isolation and treatments.

Following collection, tracheal tissue was placed in 5 mL DMEM + 1% antimycotic-antibiotic (Thermo Fisher Scientific). Primary TrFb (HTrFb) were isolated with an enzyme cocktail containing pronase (0.15% in Ham’s/F12, Sigma-Aldrich), dispase (3 mg/mL, Thermo Fisher Scientific), collagenase (2 mg/mL, Sigma-Aldrich), for 1 hour at 37°C, and DNase (0.25 mg/mL, Sigma-Aldrich) for 10 minutes. RBCs were removed by sequential treatment with RBC Lysis Buffer (eBiosciences, Thermo Fisher Scientific) for 5 minutes. Next, tracheal tissue was then passed through 100 μm, 70 μm, and 40 μm mesh filters. Filtrated cells were centrifuged (5 minutes at 500*g*) to obtain a pellet and cultured in DMEM medium (Thermo Fisher Scientific) with 10% FBS (Thermo Fisher Scientific) and 1% antimycotic-antibiotic (Thermo Fisher Scientific) and were used at passage 3. Cells were cultured at 37°C, in 5% CO_2_.

Cells were seeded and grown until 60% confluence before being treated with 5 ng/mL TGF-β1 (R&D Systems) with or without SB431542 for 24 hours at 37°C and underwent immunofluorescence staining for CTHRC1. The mean fluorescence intensity of CTHRC1 or colocalization with COL1 was measured using ImageJ and compared between treatment and nontreatment groups.

### Human Fb-basal cells cocultures.

Cells were cultured using the modified conditional reprogramming culture (mCRC) method (36). Accordingly, the cells were cocultured on a feeder layer of irradiated HTrFb with F-medium supplemented with Rho-kinase inhibitor, Y-27632 (10 μM, ApexBio). Cocultures were maintained for 5 days at 37°C in 5% CO_2_ before undergoing fixation and immunofluorescence staining for KRT5, KRT14, and VIMENTIN. The total basal cell area was measured using NIS-elements software based on KRT5^+^KRT14^+^VIM^–^DAPI^+^ cells.

### Statistics.

All data were analyzed using GraphPad Prism, version 10.1.0 (GraphPad Software Inc.). Data normality was evaluated by the Shapiro-Wilk test (S-W). The statistical significance of differences was evaluated by Mann-Whitney *U* test, 2-tailed Student’s *t* test, or 1-way or 2-way ANOVA followed by a post hoc Dunnett’s or Tukey’s multiple-comparison test, depending on the type of distribution and the number of comparison groups. Differences were considered statistically significant at *P* < 0.05. The type of analysis used for each data set and the specific sample size (*n*) for each experimental set are indicated in the legends of the corresponding figures.

### Study approval.

Human tracheal tissues were harvested under the IRBs of 2 tertiary-level pediatric hospitals (Nationwide Children’s Hospital IRB, STUDY00000847). Mouse experiments were performed according to IACUC-approved protocols at Nationwide Children’s Hospital (AR15-00090) in agreement with the Public Health Service, NIH, in the Care and Use of Laboratory Animals (2011) and US Department of Agriculture (USDA) regulations outlined in the Animal Welfare Act.

### Data availability.

Values for all data points in graphs are reported in the Supporting Data Values file. Previously published mouse scRNA-Seq data that were reanalyzed in this study are available in NCBI-SRA with the accession no. PRJNA954770.

## Author contributions

JC, ZHT, SDR, and TC participated in the conceptualization and work design. JC, ZH, ZHT, LL, SD, KMS, TAV, SDR, and TC contributed to the acquisition, analysis, and data interpretation. JC, ZH, SDR, and TC wrote the main draft of the manuscript. JC, ZH, ZHT, LL, SD, KMS CKB, TAV, SDR, and TC contributed to editing the main draft of the manuscript. JC, CKB, SDR, and TC provided scientific advice. All authors approved the final version submitted for publication.

## Supplementary Material

Supplemental data

Supporting data values

## Version 1 01/21/2025

In-Press Preview

## Version 2 03/10/2025

Electronic publication
