# Supplementary material for: Orchestrated response from heterogenous fibroblast subsets contributes to repair from surgery-induced stress after airway reconstruction
Source: JCI Insight. 2025 Jan 21;10(5):e186263. doi: 10.1172/jci.insight.186263 (PMC11949024; doi:10.1172/jci.insight.186263)
Supplement: Supplemental data [file jciinsight-10-186263-s151.pdf]

Supplemental Materials

Supplemental Figures

A

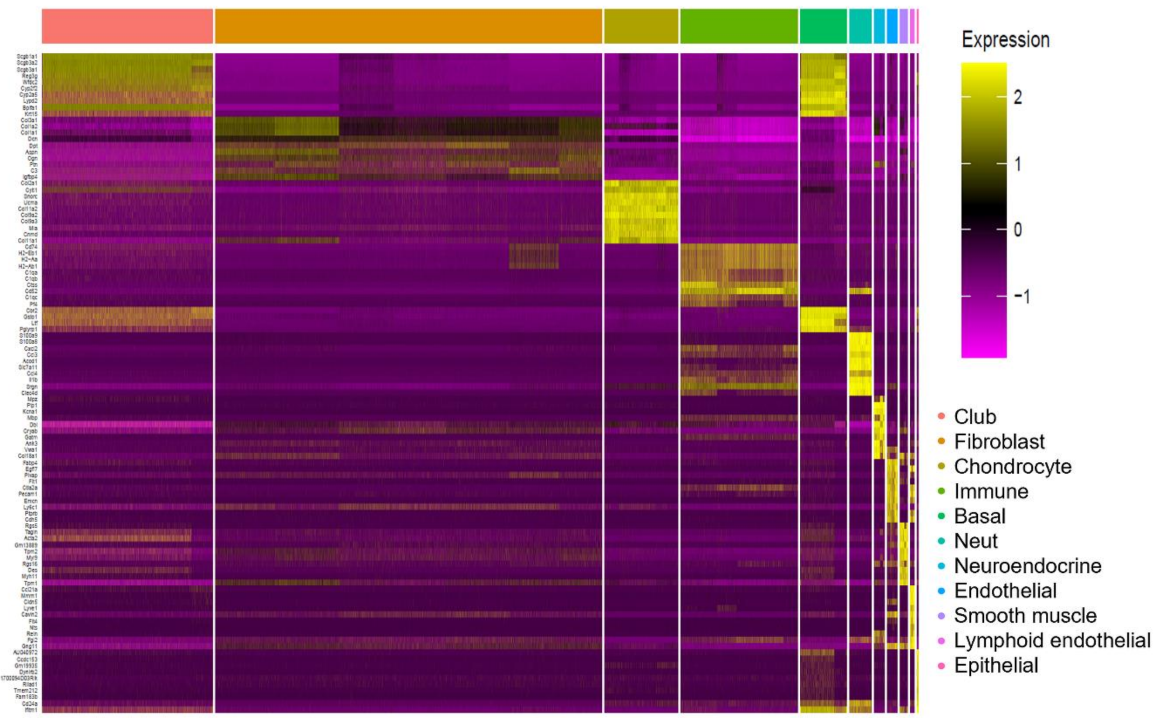

Supplemental Figure 1. Airway cell types identified by sc-RNASeq. (**A**) Heatmap showing the ten most differentially expressed genes of each cell cluster, as provided by Seurat. Each column represents a single cell, each row represents an individual gene. All marker genes per cluster are shown on the left. Yellow indicates maximum gene expression and purple indicates no expression in scaled log normalized UMI counts.

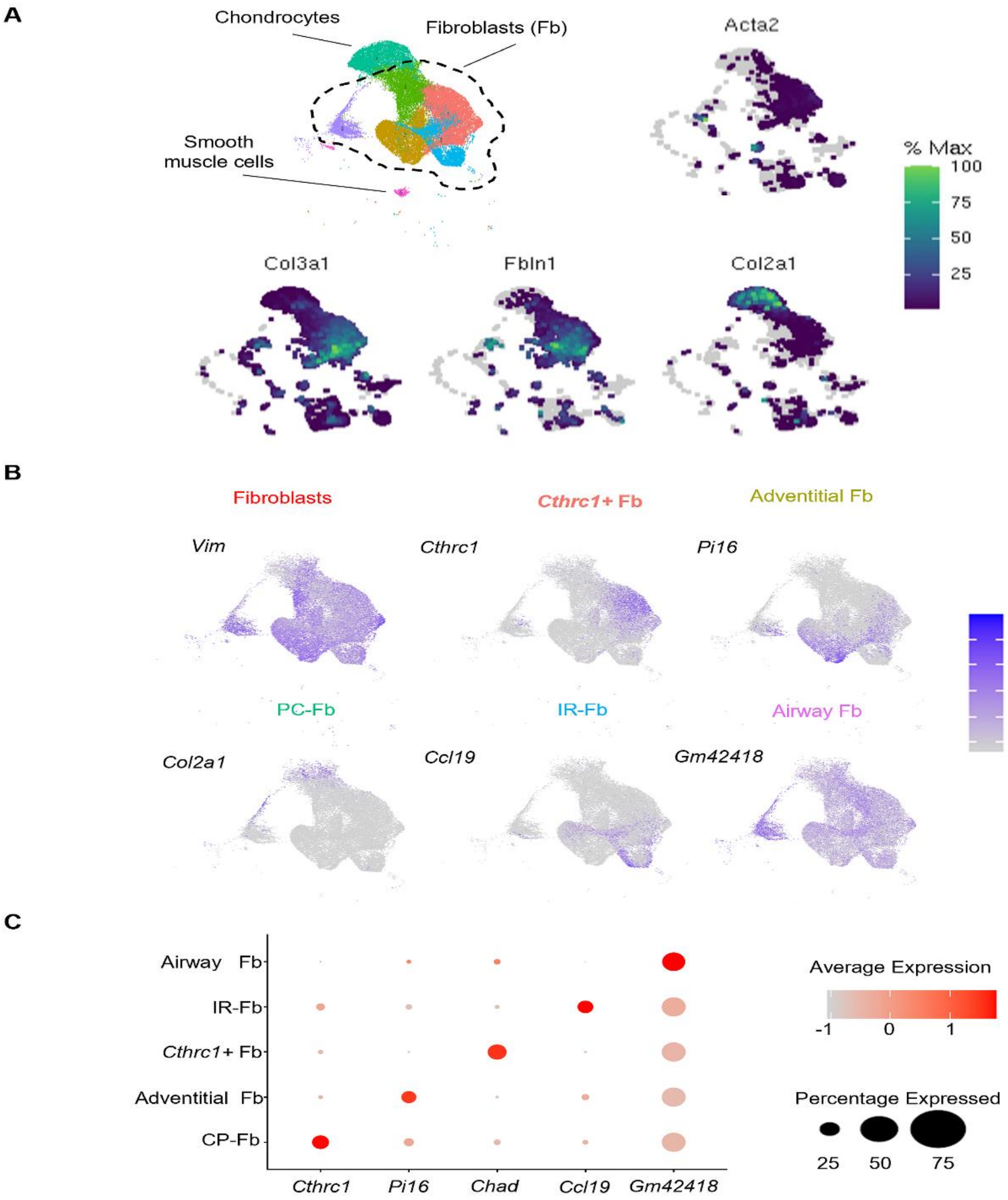

Supplemental Figure 2. Fibroblast clusters identification. (**A**) UMAP and annotations of fibroblast clusters from all conditions and mesenchymal markers gene expression plot on UMAP layout in the cells from all comparisons. (**B**) Gene expression levels of representative fibroblasts markers in a UMAP. (**C**) Dotplot shows gene expression of top fibroblasts subtype markers. Color indicates average expression and dot size percentage of cells expressing the marker.

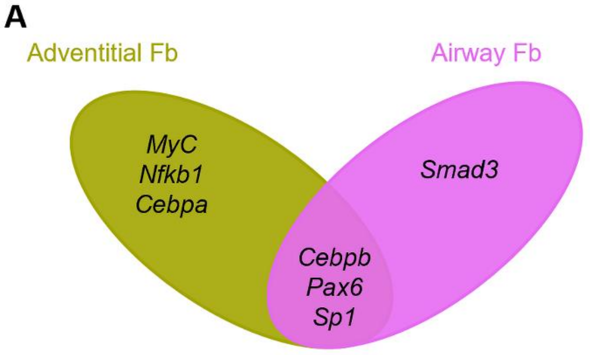

Supplemental Figure 3. Potential transcription factors regulating fibroblasts homeostasis. (**A**) Venn diagram showing identified potential transcription factors in Adventitial Fb (light green) and Airway Fb (lilac).

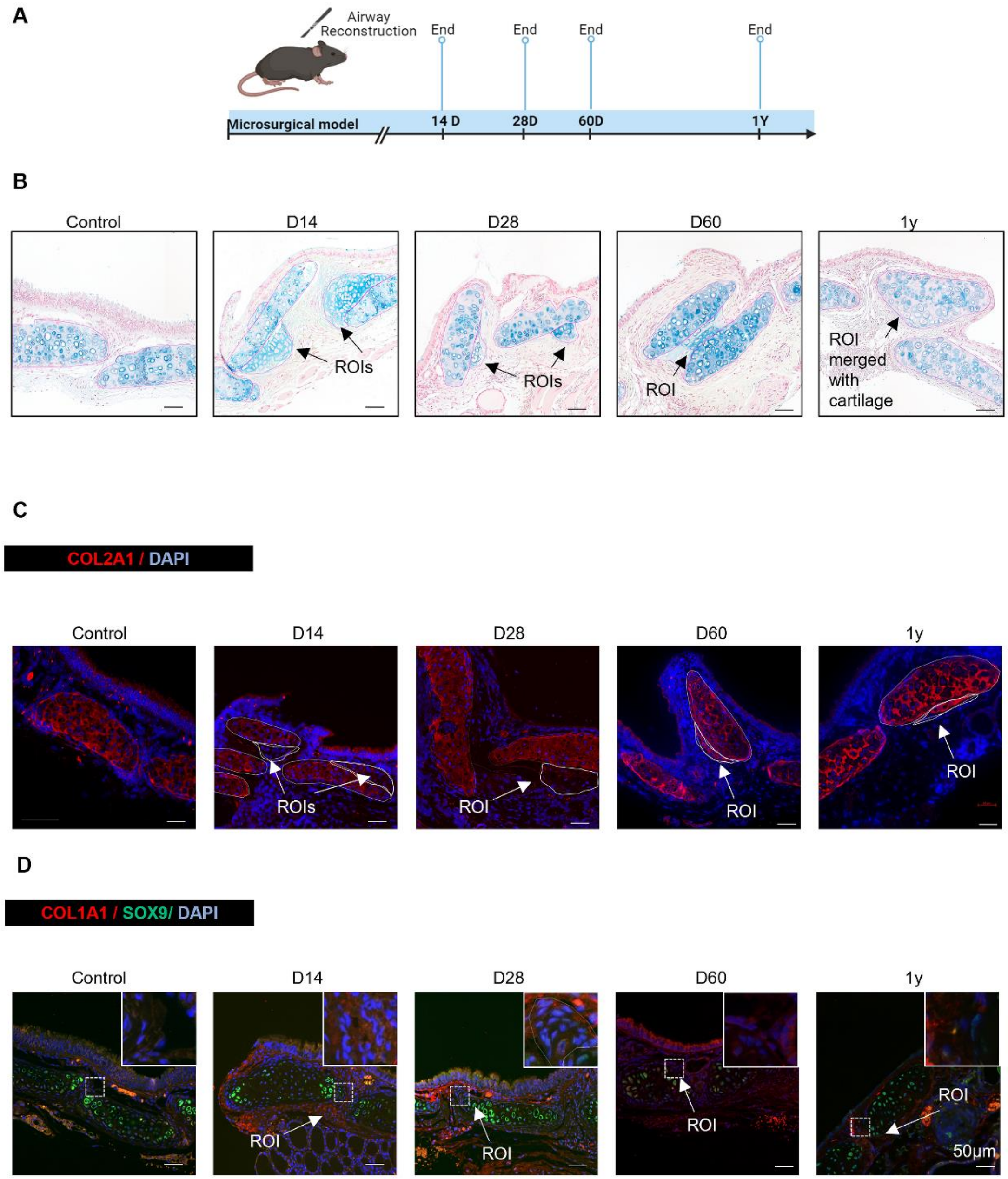

Supplemental Figure 4. Fibrocartilage-like structure characterization. (**A**) Graphical representation of the microsurgical model of airway reconstruction evaluated at homeostasis, D14, D28, D60, and 1Y post-reconstruction. (**B**) Representative images of Alcian blue positive Fibrocartilage-like structures at each time point. (**C**) Representative images of COL2 positive Fibrocartilage-like structures at each time point. (**D**) Representative images and magnifications of the region of interest of COL1/SOX9 double positive Fibrocartilage-like structures at each time point. ROI represents the Fibrocartilage-area and “C” denotes cartilage. Scale bars: 50µm.

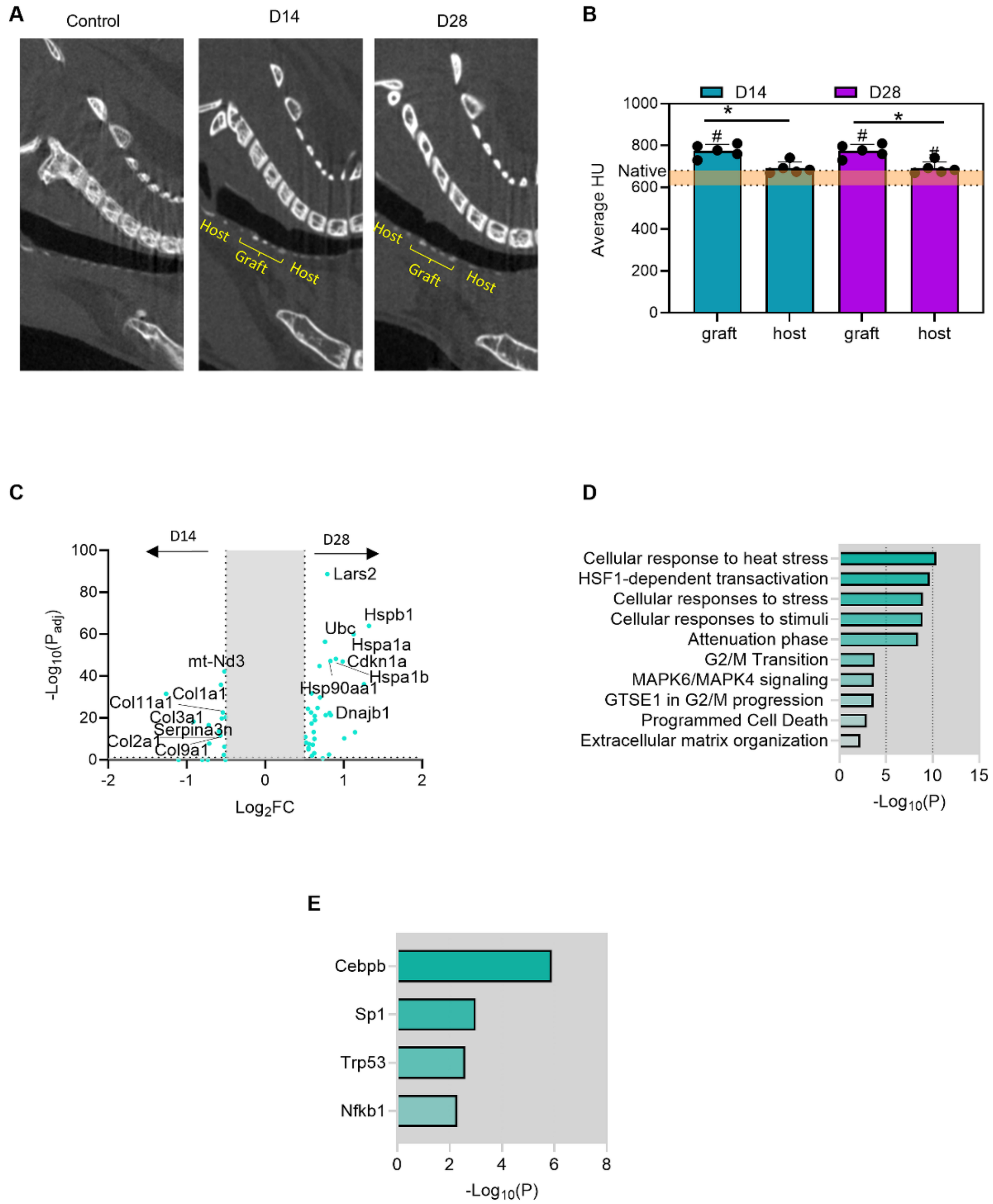

Supplemental Figure 5. Impact of SIS in the chondrocytes. (**A**) Representative sagittal reconstructions of micro-CT images. (**B**) Average Hounsfield units (HU) quantification (Native n=5, D14 n=5, D28 n=5). (**C**) Volcano plot of dysregulated expressed genes according to their statistical P-value (y-axis) and their relative abundance ratio (log2 fold change) between SIS at D28 and D14 identifying cellular stress-associated genes (*Lars2, Hspb1, Ubc, Hspa1a, Cdkn1a, Hspa1b, Hsp90aa1,* and *Dnajb1*). (**D**) GO STRING generated networks of proteins corresponding to upregulated genes in response to SIS at D28 compared with normal airways. (#) denotes a significant difference compared to the host; (*) represents significantly higher HU in the reconstructed versus normal airway. Statistical analysis was performed using 2-way ANOVA with Tukey's multiple-comparison test. Bars represent mean +/- S.D. *; #P < 0.05.

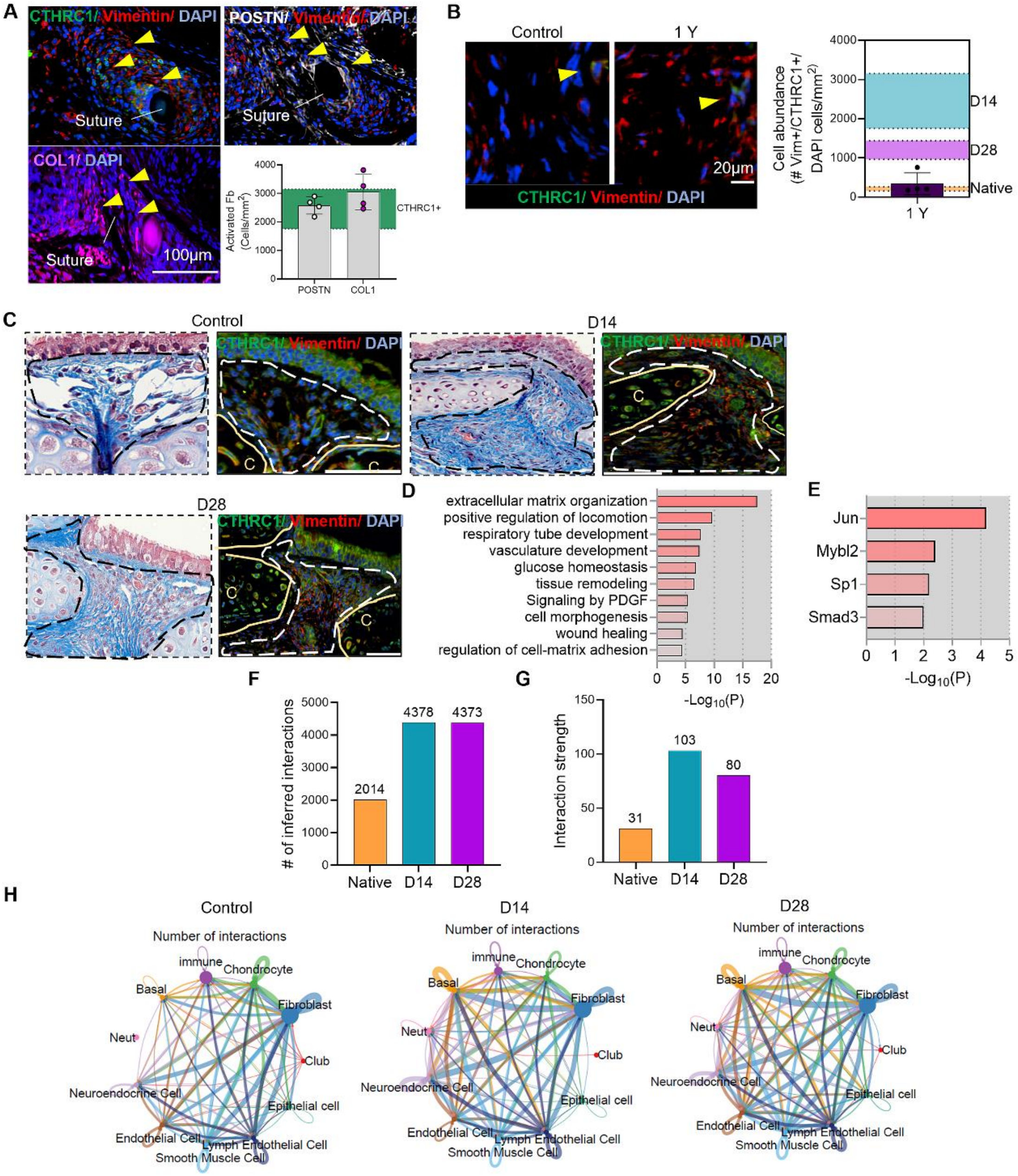

Supplemental Figure 6. *Cthrc1*+ Fb spatial distribution, gene expression, and CellChat inferred interactions. (**A**) Representative immunofluorescence staining showing the presence of activated fibroblasts using different markers (Vimentin+, red, and CTHRC1+, green, POSTN+, white, and COL1+, purple) and corresponding quantification. Scale bars: 100µm. (**B**) Representative immunofluorescence staining of *Cthrc1*+ Fb at different time points post-SIS. Scale bars: 20µm. (**C**) Representative images of dense collagen areas and their corresponding serial section staining for *Cthrc1*+ Fb (Vimentin+/CTHRC1+ double positive cells) in homeostasis, D14, and D28 post-reconstruction. Scale bars: 50µm. (**D**) GO enriched pathways corresponding to changes in *Cthrc1*+ Fb at D14. FDR < 10%. (**E**) Bar graph showing identified potential transcription factors in *Cthrc1*+ Fb at D14 compared to control. Bar graphs showing the number of inferred interactions (**F**) and interaction strength (**G**) from all the cell types per condition. (**H**) Circle dot of the number of interactions displaying each cell type during normal (left panel), D14 (middle panel), and D28 (right panel).

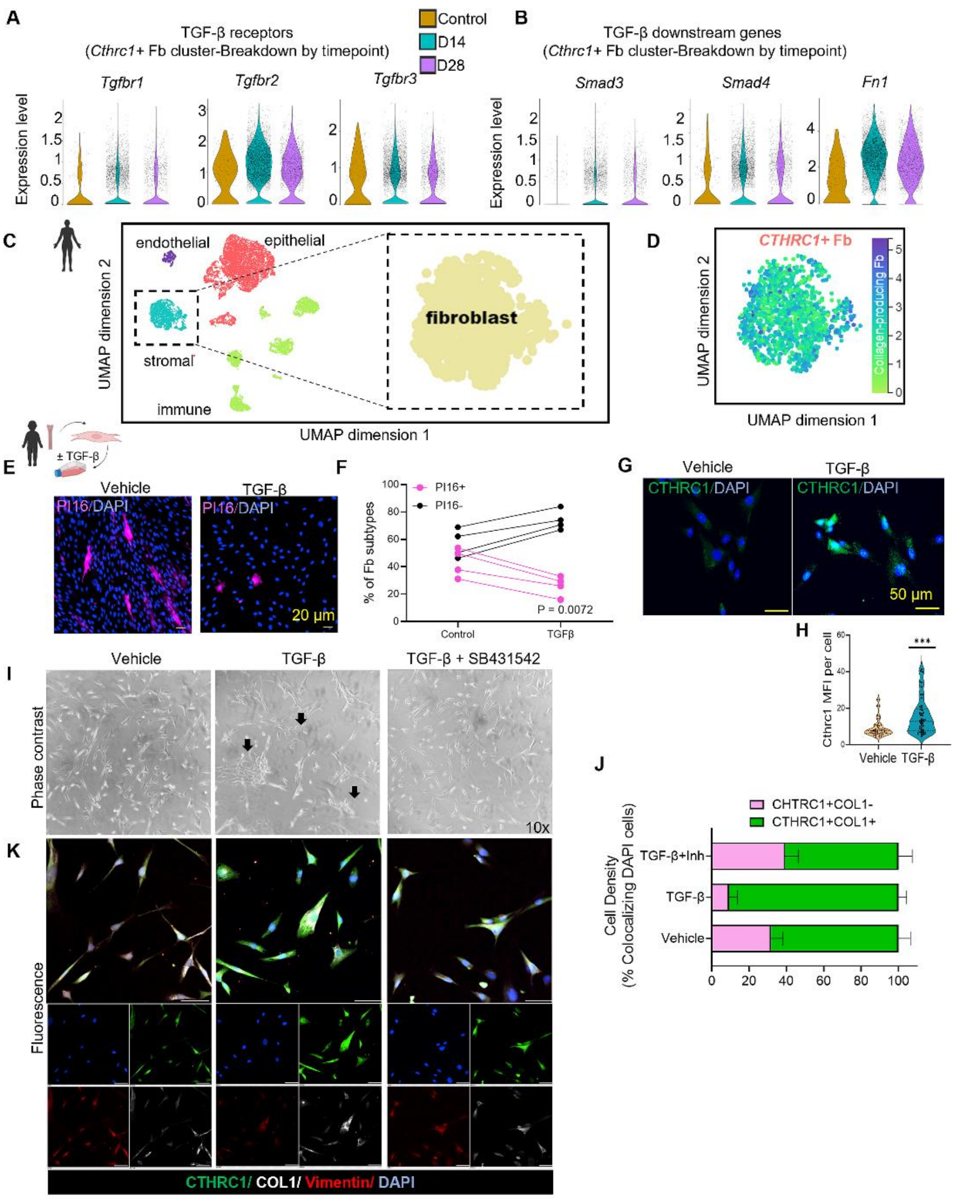

Supplemental Figure 7. TGFβ drives activation of Fb to *Cthrc1*+ Fb and modulates their collagen production. (**A**) Violin plots showing dynamic expression of TGFβ receptors *Tgfbr1, Tgfr2,* and *Tgfbr3* in *Cthrc1*+ Fb during homeostasis, D14, and D28 post-reconstruction. (**B**) Violin plots showing dynamic expression of selected genes from the TGF-β canonical pathway *Smad3, Smad4,* and *Fn1* in *Cthrc1*+ Fb during homeostasis, D14, and D28 post-reconstruction. (**C**) UMAP of all scRNAseq cells from the human normal airway, capturing fibroblast subset. (**D**) Gene set expression on a UMAP layout of the top gene markers that identified the collagen-*CTHRC1*+ Fb (cellxgene tool). Scale bars: 20µm and 50µm. (**E**) Representative immunofluorescent staining of PI16 on human isolated fibroblast culture *in vitro* for 24 hours with and without TGFβ1 and (**F**) corresponding quantification (n=4). (**G**) Representative immunofluorescent staining of CTHRC1 on human isolated fibroblast culture *in vitro* for 24 hours with and without TGFβ1 and (**H**) corresponding quantification. Violin plot of mean fluorescence intensity. (**I**) Representative images phase contrast, (**J**) stacked bar-plot denoting the relative frequency of CTHRC1+COL1-, CTHRC1+COL1- Fb, and (**K**) fluorescence images of human isolated fibroblasts culture *in vitro* for 24 hours in the presence of TGFβ1 and with or without TGFβ type I receptor inhibitor (SB431542) Scale bars: 50µm. Statistical analysis was performed using paired t test (**F**) and Mann-Whitney test (**H**). ***P < 0.001.

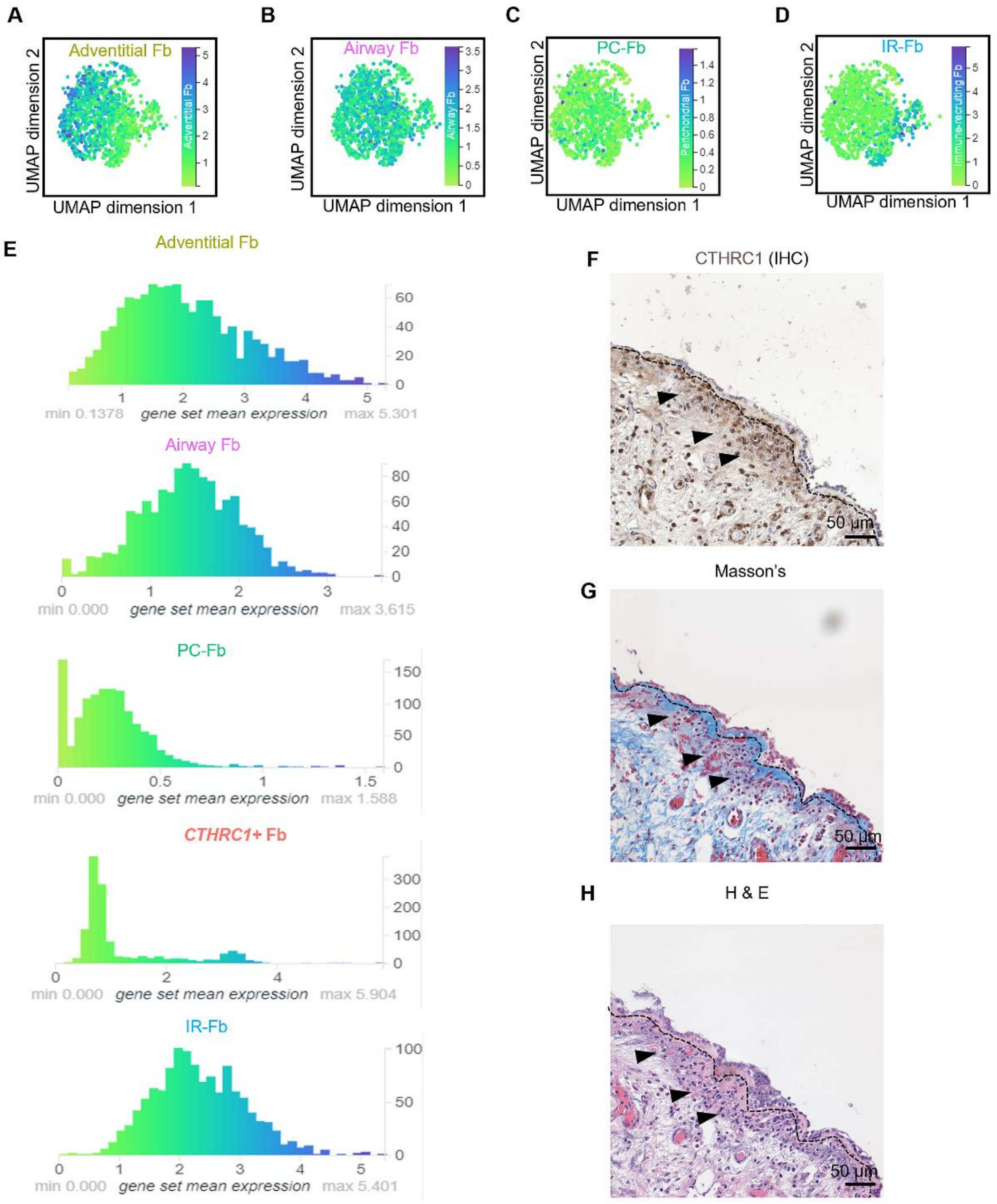

Supplemental Figure 8. Identification of fibroblast subsets in the normal human airway. Gene set expression on a UMAP layout of the top gene markers that identified the adventitial Fb (**A**), airway Fb (**B**), perichondrial Fb (**C**), and immune-recruiting Fb (**D**) cellxgene tool and (**E**) corresponding histograms. Representative image of the human airway from a pediatric patient that underwent airway reconstruction (**F**) CTHRC1 immunostaining, (**G**) H&E, and (**H**) Masson's trichrome staining. Black triangles indicate submucosal cells expressing CTHRC1. The dotted line delineates the epithelium from the submucosa. Scale bars: 50µm.

Supplemental Tables

Supplemental Table 1. Pediatric patient demographics.

	Pediatric patients (N=6)
Age (years [SE])	7.2 years [3.5]
Sex	
Male	4 (66.67%)
Female	2 (33.33%)

Supplemental Table 2. Collagens (Matrisome category).

**Gene Symbol**	**Gene Name**
Col1a1	Collagen, type I, alpha 1
Col1a2	Collagen, type I, alpha 2
Col2a1	Collagen, type II, alpha 1
Col3a1	Collagen, type III, alpha 1
Col4a1	Collagen, type IV, alpha 1
Col4a2	Collagen, type IV, alpha 2
Col4a3	Collagen, type IV, alpha 3
Col5a1	Collagen, type V, alpha 1
Col5a2	Collagen, type V, alpha 2
Col6a1	Collagen, type VI, alpha 1
Col6a2	Collagen, type VI, alpha 2
Col6a3	Collagen, type VI, alpha 3
Col7a1	Collagen, type VII, alpha 1
Col8a1	Collagen, type VIII, alpha 1
Col9a1	Collagen, type IX, alpha 1
Col9a2	Collagen, type IX, alpha 2
Col10a1	Collagen, type X, alpha 1
Col11a1	Collagen, type XI, alpha 1
Col12a1	Collagen, type XII, alpha 1
Col13a1	Collagen, type XIII, alpha 1
Col14a1	Collagen, type XIV, alpha 1
Col15a1	Collagen, type XV, alpha 1
Col16a1	Collagen, type XVI, alpha 1
Col18a1	Collagen, type XVIII, alpha 1
Col20a1	Collagen, type XX, alpha 1
